# Comprehensive analysis of the effect of rs2295080 and rs2536 polymorphisms within the *mTOR* gene on cancer risk

**DOI:** 10.1042/BSR20191825

**Published:** 2020-07-09

**Authors:** Guang-Hui Qi, Chun-Hui Wang, Hong-Ge Zhang, Jian-Guo Yu, Fei Ding, Zhi-Chao Song, Qing-Hua Xia

**Affiliations:** 1 Department of Urology, The First Hospital of Zibo City, Zibo, Shandong 255000, China; 2 Second Department of Gastroenterology, The First Hospital of Zibo City, Zibo, Shandong 255000, China; 3 Third Department of Surgery, Teng zhou Hospital of Traditional Chinese Medicine, Teng zhou, Shandong 277500, China; 4 Second Department of Oncology, The First Hospital of Zibo City, Zibo, Shandong 255000, China; 5 Department of Anorectal Surgery, The First Hospital of Zibo City, Zibo, Shandong 255000, China; 6 Department of Urology, Shandong Provincial Hospital, Cheeloo College of Medicine, Shandong University, Jinan, Shandong 250021, China

**Keywords:** cancer, mTOR, polymorphism, risk

## Abstract

There is still no conclusion on the potential effect of the rs2295080 and rs2536 polymorphisms of *mTOR* (mammalian target of rapamycin) gene on different cancers. Herein, we performed a comprehensive assessment using pooled analysis, FPRP (false-positive report probability), TSA (trial sequential analysis), and eQTL (expression quantitative trait loci) analysis. Eighteen high-quality articles from China were enrolled. The pooled analysis of rs2295080 with 9502 cases and 10,965 controls showed a decreased risk of urinary system tumors and specific prostate cancers [TG vs. TT, TG+GG vs. TT and G vs. T; *P*<0.05, OR (odds ratio) <1]. FPRP and TSA data further confirmed these results. There was an increased risk of leukemia [G vs. T, GG vs. TT, and GG vs. TT+TG genotypes; *P*<0.05, OR>1]. The eQTL data showed a potential correlation between the rs2295080 and *mTOR* expression in whole blood samples. Nevertheless, FPRP and TSA data suggested that more evidence is required to confirm the potential role of rs2295080 in leukemia risk. The pooled analysis of rs2536 (6653 cases and 7025 controls) showed a significant association in the subgroup of “population-based” control source via the allele, heterozygote, dominant, and carrier comparisons (*P*<0.05, OR>1). In conclusion, the TG genotype of *mTOR* rs2295080 may be linked to reduced susceptibility to urinary system tumors or specific prostate cancers in Chinese patients. The currently data do not strongly support a role of rs2295080 in leukemia susceptibility. Large sample sizes are needed to confirm the potential role of rs2536 in more types of cancer.

## Introduction

Considering the involvement of genetic and environmental factors in tumorigenesis [1,2], it is very informative to discover cancer-associated SNPs (single-nucleotide polymorphisms) [3]. The inconclusive roles of SNPs in specific cancer types suggest that pooled analysis is warranted. A meta-analysis containing 11,204 subjects reported that the rs699947 polymorphism within the *VEGF* (vascular endothelial growth factor) gene was associated with an increased risk of bladder cancer and renal cell carcinoma in Asians [4]. Another meta-analysis with 34,911 cases and 48,329 controls showed the genetic relationship between the *BRCA2* (BRCA2 DNA repair associated) rs144848 polymorphism and the overall risk of cancer [5].

The human *mTOR* (mammalian target of rapamycin) gene, also called *FRAP* (FKBP12 rapamycin-associated protein), functions as an essential serine-threonine kinase during signal transduction and is involved in the biological processes of cellular proliferation, cell cycle, cell motility, cell survival, or autophagy [6,7]. The abnormal function of mTOR signaling is thought to be associated with oncogenesis [8–10]. Inhibition of the PI3K (phosphatidylinositol 3-kinase)/AKT/mTOR signaling pathway is employed in therapeutic approaches for certain cancer types [11]. Two polymorphisms, rs2295080 and rs2536, have been identified in the human *mTOR* gene, mapping to chromosome 1p36.22 [12–15]. In the present study, we are interested in evaluating the possible effect of the two polymorphisms on the susceptibility to different cancers through a series of analyses.

Unlike four previously reported meta-analyses [13–16], this meta-analysis features newly published articles, and we utilized a different strategy for a comprehensive analysis. Three factors, including cancer type, genotyping method and control source, were considered for the subgroup analyses. Importantly, we performed FPRP analysis, TSA, and eQTL analysis to assess pooled data and the correlation between genotype and gene expression.

## Methods

### Study selection

We retrieved studies from four online databases (updated to April 2020), PubMed, Embase (Excerpta Medica database), Cochrane, and WANFANG. Supplementary Table S1 presents our main search terms. Next, we screened the obtained articles, referring to the guidelines of PRISMA (Preferred Reporting Items for Systematic Reviews and Meta-analyses) [17], and relevant publications [18,19]. Selection factors included overlapping or duplicated data; reviews, case reports, and trials; cellular or animal assays; conference abstracts; meta-analyses; and other diseases, genes or SNPs. The genotype frequency distribution in controls was required to follow HardyñWeinberg equilibrium (HWE). The genotype frequency data of the *mTOR* gene rs2295080 and rs2536 polymorphisms in both cancer cases and negative controls needed to be extractable from the studies.

### Information extraction

We extracted the information independently and utilized a table to summarize the following features: first author name, year of publication, genotypic/allelic frequency, cancer type, source of control, genotyping method, and sample size. We also evaluated the methodological quality of each article with a quality score, as reported previously [20,21]. When the quality score was >9, the study was considered high quality.

### Pooled analysis

The ORs (odds ratios), 95% CIs (confidence intervals), and *P*_Association_ values (*P* values of the association test) were calculated to evaluate association strength and properties. Six genotype comparisons, namely, allele (allele (G vs. T) for rs2295080; allele (C vs. T) for rs1536), homozygote (GG vs. TT; CC vs. TT), heterozygote (TG vs. TT; TC vs. TT), dominant (TG+GG vs. TT; TC+CC vs. TT), recessive (GG vs. TT+TG; CC vs. TT+TC), and carrier (carrier (G vs. T); carrier (C vs. T)) comparisons, were used. An overall meta-analysis and subsequent subgroup analyses according to three factors (control source, genotyping method, and cancer type) were conducted. A random-effects model was used when *I*^2^ > 50.0% or *P*_heterogeneity_ (*P* value of the heterogeneity) < 0.05. When using Egger’s/Begg’s tests, *P*_Egger_ (*P* value of Egger’s test) <0.05 and *P*_Begg_ (*P* value of Begg’s test) <0.05 indicate the presence of large publication bias. A stable OR value during sensitivity analysis reflects the robustness of the result to a certain extent. Stata software (StataCorp LP, U.S.A.) was used for the above analyses.

### FPRP analysis

We also performed false-positive report probability (FPRP) analysis on the positive data from the pooled analyses, as described previously [22,23]. The chi-square test was adopted for the evaluation of the genotype frequency distributions. Statistical power was also determined. Six prior probability levels (0.25, 0.1, 0.01, 0.001, 0.0001, and 0.00001) were applied. A noteworthy association was considered when the FPRP value was less than 0.2 at a prior probability of 0.01

### TSA test

To further confirm the robustness of the conclusions, we conducted trial sequential analysis (TSA), as described previously [19,24]. TSA viewer software (Copenhagen Trial Unit, Copenhagen) was employed to generate a TSA plot with the required information size (RIS) line and TSA monitoring boundaries with a type I error limit of 5% and a statistical power of 80%.

### eQTL analysis

We also utilized datasets of the GTEx (The Genotype-Tissue Expression) project (http://www.gtexportal.org/home/) [25,26] to perform an expression quantitative trait loci (eQTL) analysis to predict the correlation between the rs2295080 and rs2536 SNPs and the expression level of the *mTOR* gene (ENSG00000198793.12). Considering the above pooled data, two cell samples (EBV_transformed_lymphocytes and cultured_fibroblasts) and specific tissue samples (esophagus, stomach, and prostate) or blood samples (whole blood) were analyzed. The eQTL violin plots are provided.

## Results

### Study selection

**Figure 1:**
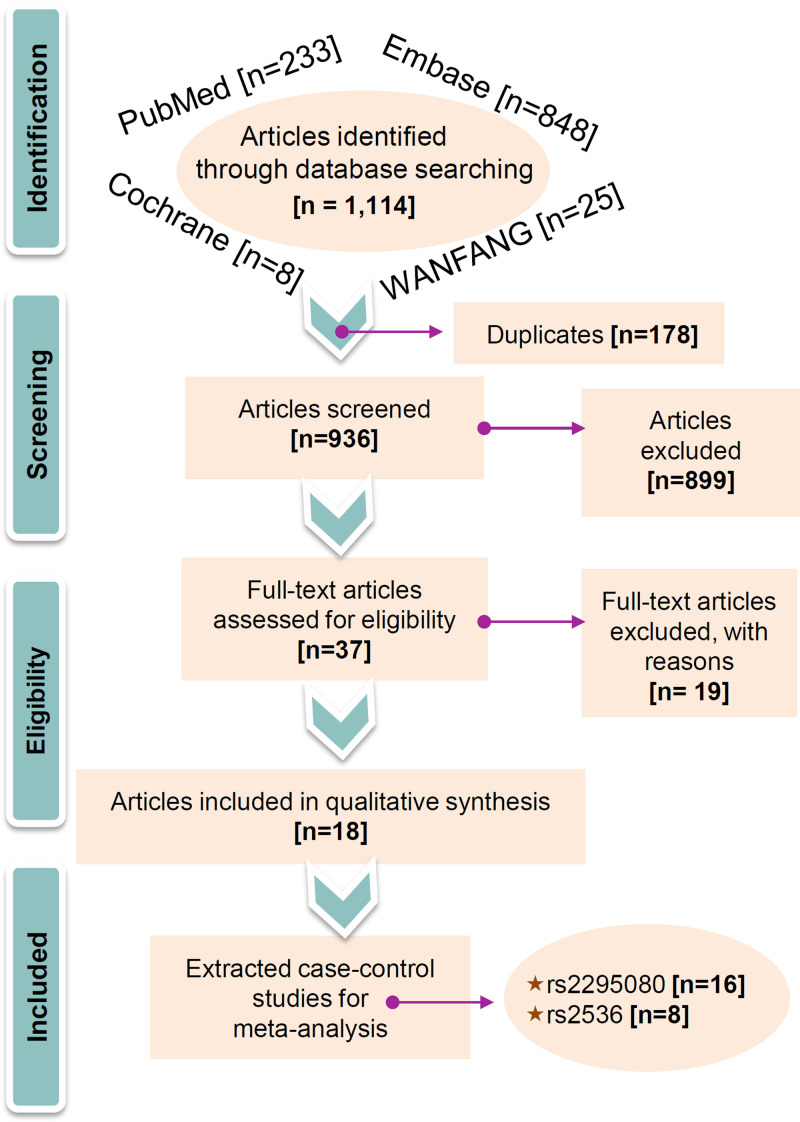
Flow chart of eligible article selection

Briefly, in total, 1114 articles were retrieved from three databases. Among them, 178 articles were first excluded due to duplicated data, and 936 articles were removed due to our exclusion criteria. Then, we obtained 37 full-text articles for evaluating eligibility and ruled out 19 ineligible articles because they lacked full genotype data in both cases and controls and did not conform to HWE. Finally, a total of 18 articles [16,27–43] from the Chinese population were selected. Of them, 16 case–control studies were pooled for the meta-analysis of rs2295080, while 8 case–control studies were pooled for the meta-analysis of rs2536. We show our detailed study diagram in Figure 1 and list the extracted information in Table 1. All the included studies were of high quality; that is, all quality assessment scores were greater than nine (Supplementary Table S2).

**Table 1:** Basic information from eligible case–control studies

First author, year	SNP	Case					Cancer type	Control					Source	Genotyping method
		MM	Mm	mm	M	m		MM	Mm	mm	M	m		
**Cao, 2012**	rs2295080	454	218	38	1126	294	RCC	438	277	45	1153	367	HB	TaqMan
	rs2536	607	99	4	1313	107	RCC	628	128	4	1384	136	HB	TaqMan
**Chen, 2012**	rs2295080	429	209	28	1067	265	Prostate cancer	413	259	36	1085	331	HB	TaqMan
	rs2536	565	96	5	1226	106	Prostate cancer	585	119	4	1289	127	HB	TaqMan
**Chen, 2019**	rs2295080	310	201	19	821	239	Breast cancer	245	198	37	688	272	PB	TaqMan
**He, 2013**	rs2536	938	179	8	2055	195	Gastric cancer	1019	170	7	2208	184	PB	TaqMan
**Huang, 2012**	rs2295080	254	140	23	648	186	ALL	353	180	21	886	222	HB	TaqMan
	rs2536	346	65	6	757	77	ALL	448	103	3	999	109	HB	TaqMan
**Li, 2013**	rs2295080	653	311	40	1617	391	Prostate cancer	617	382	52	1616	486	PB	TaqMan
	rs2536	804	192	8	1800	208	Prostate cancer	894	147	10	1935	167	PB	TaqMan
**Liu, 2017**	rs2295080	236	145	32	617	209	Prostate cancer	454	316	37	1224	390	HB	TaqMan
**Liu, 2014**	rs2536	849	186	13	1884	212	HCC	850	188	14	1888	216	HB	TaqMan
**Qi, 2017**	rs2295080	194	279	101	667	481	Gastric cancer	297	441	174	1035	789	HB	TaqMan
**Wang, 2015**	rs2295080	568	394	40	1530	474	Gastric cancer	607	355	41	1569	437	HB	TaqMan
**Wen, 2017**	rs2295080	366	170	24	902	218	Thyroid cancer	295	176	29	766	234	PB	TaqMan
**Xu, 2015**	rs2295080	482	225	30	1189	285	Colorectal cancer	459	273	45	1191	363	HB	TaqMan
**Xu, 2013**	rs2295080	482	246	25	1210	296	Gastric cancer	497	305	52	1299	409	HB	TaqMan
**Zhao, 2017**	rs2295080	178	90	15	446	120	Gastric cancer	174	86	11	434	108	PB	TaqMan
**Zhao, 2015**	rs2295080	68	50	15	186	80	ALL	173	111	12	457	135	HB	PCR-RFLP
		27	14	6	68	26	AML	173	111	12	457	135	HB	PCR-RFLP
**Zhao, 2016**	rs2295080	351	197	12	899	221	Breast cancer	345	212	26	902	264	HB	Sequenom Massarray
	rs2536	453	100	7	1006	114	Breast cancer	486	93	4	1065	101	HB	Sequenom Massarray
**Zhu, 2015**	rs2295080	674	390	49	1738	488	ESCC	702	362	49	1766	460	PB	TaqMan
**Zhu, 2013**	rs2536	951	165	7	2067	179	ESCC	957	157	7	2071	171	PB	NR

Abbreviations: ALL, acute lymphoblastic leukemia; AML, acute myeloid leukemia; ESCC, esophageal squamous cell cancer; HB, hospital-based; HCC, hepatocellular carcinoma; M, major allele (T allele for rs2295080; T allele for rs2536); m, minor allele (G allele for rs2295080; C allele for rs2536); NOS, Newcastle–Ottawa Scale; NR, not reported; PB, population-based; PCR-RFLP, polymerase chain reaction-restriction fragment length polymorphism; RCC, renal cell cancer; SNP, single-nucleotide polymorphism.

### Pooled analysis of rs2295080

An overall meta-analysis of rs2295080 with 16 case–control studies (9502 cases and 10,965 controls) from the Chinese population was first conducted. As shown in Table 2, a reduced susceptibility to cancer was observed in cases compared with controls via three of the genotype comparisons [heterozygote, *P*_Association_ = 0.017, OR (95% CIs) = 0.90 (0.83–0.98); dominant, *P*_Association_ = 0.031, OR (95% CIs) = 0.90 (0.82–0.99); carrier, *P*_Association_ = 0.009, OR (95% CIs) = 0.93 (0.89–0.98)] but not in the others.

**Table 2:** Pooling analysis of *mTOR rs2295080* A/G polymorphism

Overall/Subgroup	Result	Allele	Homozygote	Heterozygote	Dominant	Recessive	Carrier
**Overall**	OR (95% CIs)	0.93 (0.85–1.01)	0.90 (0.72–1.14)	0.90 (0.83–0.98)	0.90 (0.82–0.99)	0.94 (0.76–1.16)	0.93 (0.89–0.98)
	*P*_Association_	0.086	0.393	0.017	0.031	0.554	0.009
	Study	16	16	16	16	16	16
	[Case/control]	[9502/10,965]	[9502/10,965]	[9502/10,965]	[9502/10,965]	[9502/10,965]	[9502/10,965]
**PB**	OR (95% CIs)	0.88 (0.75–1.04)	0.76 (0.53–1.08)	0.89 (0.74–1.06)	0.87 (0.72–1.05)	0.79(0.58–1.08)	0.92 (0.84–1.00)
	*P*_Association_	0.129	0.125	0.178	0.148	0.137	0.054
	Study	5	5	5	5	5	5
	[Case/control]	[3490/3415]	[3490/3415]	[3490/3415]	[3490/3415]	[3490/3415]	[3490/3415]
**HB**	OR (95% CIs)	0.95 (0.85–1.06)	0.99 (0.73–1.34)	0.91 (0.82–1.01)	0.92 (0.82–1.03)	1.02 (0.77–1.36)	0.94 (0.88–1.00)
	*P*_Association_	0.347	0.941	0.063	0.133	0.885	0.065
	Study	11	11	11	11	11	11
	[Case/control]	[6012/7550]	[6012/7550]	[6012/7550]	[6012/7550]	[6012/7550]	[6012/7550]
**TaqMan**	OR (95% CIs)	0.91 (0.83–0.99)	0.84 (0.69–1.03)	0.89 (0.81–0.98)	0.89 (0.80–0.98)	0.88 (0.73–1.05)	0.93 (0.88–0.98)
	*P*_Association_	0.027	0.096	0.023	0.021	0.162	0.007
	Study	13	13	13	13	13	13
	[Case/control]	[8762/9790]	[8762/9790]	[8762/9790]	[8762/9790]	[8762/9790]	[8762/9790]
**Urinary system tumor**	OR (95% CIs)	0.86 (0.76–0.98)	0.92 (0.63–1.33)	0.79 (0.71–0.88)	0.80 (0.72–0.89)	1.00 (0.72–1.42)	0.87 (0.79–0.96)
	*P*_Association_	0.019	0.654	<0.001	<0.001	0.991	0.006
	Study	4	4	4	4	4	4
	[Case/control]	[2793/3326]	[2793/3326]	[2793/3326]	[2793/3326]	[2793/3326]	[2793/3326]
**Prostate cancer**	OR (95% CIs)	0.88 (0.74–1.04)	0.96 (0.57–1.62)	0.80 (0.70–0.90)	0.82 (0.71–0.94)	1.04 (0.63–1.71)	0.88 (0.79–0.99)
	*P*_Association_	0.140	0.882	<0.001	0.004	0.881	0.027
	Study	3	3	3	3	3	3
	[Case/control]	[2083/2566]	[2083/2566]	[2083/2566]	[2083/2566]	[2083/2566]	[2083/2566]
**leukemia**	OR (95% CIs)	1.24 (1.05–1.47)	2.25 (1.33–3.82)	1.07 (0.86–1.13)	1.17 (0.95–1.44)	2.25 (1.30–3.91)	1.14 (0.94–1.39)
	*P*_Association_	0.013	0.003	0.574	0.142	0.004	0.168
	Study	3	3	3	3	3	3
	[Case/control]	[597/1146]	[597/1146]	[597/1146]	[597/1146]	[597/1146]	[597/1146]
**Digestive system tumor**	OR (95% CIs)	0.95 (0.83–1.08)	0.84 (0.65–1.08)	0.96 (0.85–1.13)	0.96 (0.82–1.12)	0.85 (0.69–1.05)	0.97 (0.90–1.05)
	*P*_Association_	0.443	0.175	0.773	0.598	0.126	0.480
	Study	6	6	6	6	6	6
	[Case/control]	[4462/4930]	[4462/4930]	[4462/4930]	[4462/4930]	[4462/4930]	[4462/4930]
**Gastric cancer**	OR (95% CIs)	0.96 (0.81–1.14)	0.85 (0.60–1.21)	1.00 (0.84–1.18)	0.97 (0.80–1.18)	0.85 (0.63–1.10)	0.98 (0.89–1.08)
	*P*_Association_	0.649	0.364	0.970	0.799	0.299	0.647
	Study	4	4	4	4	4	4
	[Case/control]	[2612/3040]	[2612/3040]	[2612/3040]	[2612/3040]	[2612/3040]	[2612/3040]

Abbreviations: CI, confidence interval; HB, hospital-based; OR, odds ratio; PB, population-based.

Subgroup analyses according to three factors (control source, genotyping assay, and cancer type) were then conducted. As shown in Table 2, we observed positive results with the allele, heterozygote, dominant, and carrier comparisons in the subgroup of studies employing “TaqMan” analysis (all OR<1, *P*_Association_<0.05) but not in the subgroups analysis by control source.

Similarly, we observed a decreased risk of urinary system tumors via the allele [allele (G vs. T), *P*_Association_ = 0.019, OR (95% CIs) = 0.86 (0.76–0.98)], heterozygote [TG vs. TT, *P*_Association_<0.001, OR (95% CIs) = 0.79 (0.71–0.88)], dominant [TG+GG vs. TT, *P*_Association_<0.001, OR (95% CIs) = 0.80 (0.72–0.89)], and carrier [carrier (G vs. T), *P*_Association_ = 0.006, OR (95% CIs) = 0.80 (0.72–0.89)] comparisons (Table 2). Positive results were observed for prostate cancer via the heterozygote [TG vs. TT, *P*_Association_<0.001, OR (95% CIs) = 0.80 (0.70–0.90)], dominant [TG+GG vs. TT, *P*_Association_ = 0.004, OR (95% CIs) = 0.82 (0.71–0.94)], and carrier [carrier (G vs. T), *P*_Association_ = 0.027, OR (95% CIs) = 0.88 (0.79–0.99)] comparisons (Table 2). These results indicated that the TG genotype of *mTOR* rs2295080 is likely to be associated with a decreased susceptibility to urinary system tumors and specific prostate cancers in Chinese patients. However, we detected negative results in the subgroup of studies on digestive system tumors and specific gastric cancers (Table 2, all *P*_Association_>0.05).

**Figure 2:**
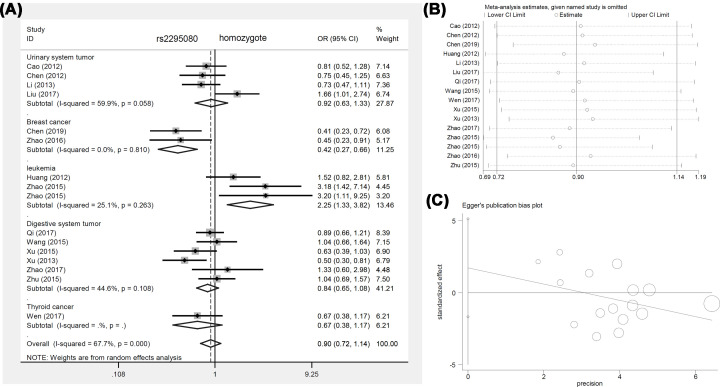
Pooled analysis of *mTOR* rs2295080 via the homozygote comparison (**A**) Forest plot of the subgroup analysis by cancer type. (**B**) Begg’s test. (**C**) Sensitivity analysis.

**Figure 3:**
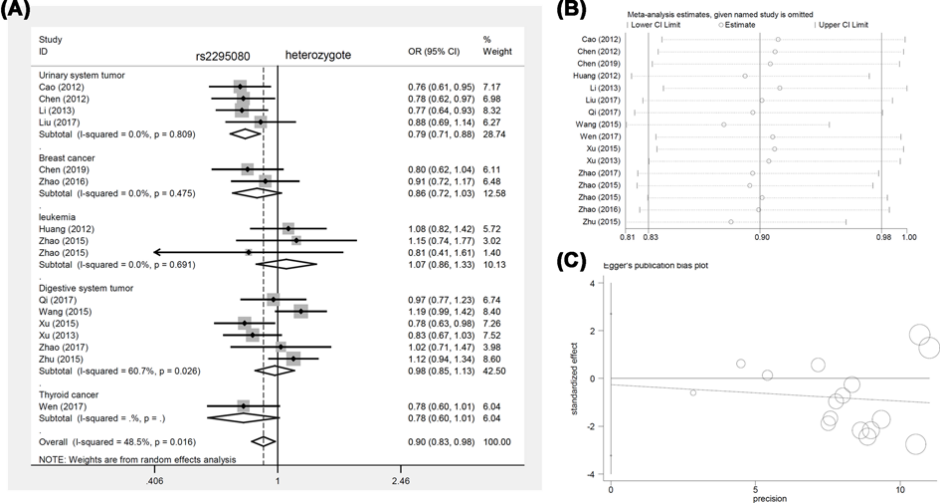
Pooling analysis of *mTOR* rs2295080 under the heterozygote model (**A**) Forest plot of subgroup analysis by cancer type. (**B**) Begg’s test. (**C**) Sensitivity analysis data.

Interestingly, we observed an increased risk for leukemia in cases in the allele [allele (G vs. T), *P*_Association_ = 0.013, OR (95% CIs) = 1.24 (1.05–1.47)], homozygote [GG vs. TT, *P*_Association_ = 0.003, OR (95% CIs) = 2.25 (1.33–3.82)], and recessive [GG vs. TT+TG, *P*_Association_ = 0.004, OR (95% CIs) = 2.25 (1.30–3.91)] comparisons, suggesting a potential relationship between the GG genotype of *mTOR* rs2295080 and an increased leukemia risk in the Chinese population. We present the forest plot data of the subgroup analysis by disease type in Figure 2A (homozygote comparison), Figure 3A (heterozygote comparison), Supplementary Figure S1A (allele comparison), Supplementary Figure S2A (dominant comparison), Supplementary Figure S3A (recessive comparison), and Supplementary Figure S4A (carrier comparison). We also present the forest plot data of the subgroup analysis of *mTOR* rs2295080 by control source (Supplementary Figure S5) and genotype method (Supplementary Figure S6).

### Pooled analysis of rs2536

A total of eight case–control studies with 6653 cases and 7025 controls were included in the pooled analysis of rs2536. As shown in Table 3, there was a significant association in the subgroup of studies using “PB” as a control source in the allele [allele (G vs. A), *P*_Association_ = 0.012, OR (95% CIs) = 1.17 (1.04–1.32)], heterozygote [AG vs. AA, *P*_Association_ = 0.047, OR (95% CIs) = 1.21 (1.00–1.45)], dominant [AG+GG vs. AA, *P*_Association_ = 0.038, OR (95% CIs) = 1.20 (1.01–1.42)], and carrier [carrier (G vs. A), *P*_Association_ = 0.023, OR (95% CIs) = 1.16 (1.02–1.32)] comparisons. However, we observed negative results in other comparisons (Table 3, all *P*_Association_>0.05).

**Table 3:** Pooling analysis of *mTOR rs2536* A/G polymorphism

Overall/Subgroup	Result	Allele	Homozygote	Heterozygote	Dominant	Recessive	Carrier
**Overall**	OR (95% CIs)	1.05 (0.97–1.14)	1.16 (0.80–1.69)	1.03 (0.89–1.18)	1.04 (0.91–1.18)	1.15 (0.80–1.68)	1.04 (0.95–1.14)
	*P*_Association_	0.252	0.424	0.722	0.604	0.450	0.364
	Study	8	8	8	8	8	8
	[Case/control]	[6653/7025]	[6653/7025]	[6653/7025]	[6653/7025]	[6653/7025]	[6653/7025]
**PB**	OR (95% CIs)	1.17 (1.04–1.32)	1.03 (0.58–1.82)	1.21 (1.00–1.45)	1.20 (1.01–1.42)	0.99 (0.56–1.76)	1.16 (1.02–1.32)
	*P*_Association_	0.012	0.928	0.047	0.038	0.983	0.023
	Study	3	3	3	3	3	3
	[Case/control]	[3252/3368]	[3252/3368]	[3252/3368]	[3252/3368]	[3252/3368]	[3252/3368]
**HB**	OR (95% CIs)	0.96 (0.85–1.07)	1.28 (0.78–2.09)	0.92 (0.81–1.05)	0.93 (0.82–1.06)	1.29 (0.79–2.11)	0.95 (0.84–1.07)
	*P*_Association_	0.445	0.331	0.203	0.296	0.312	0.382
	Study	5	5	5	5	5	5
	[Case/control]	[3401/3657]	[3401/3657]	[3401/3657]	[3401/3657]	[3401/3657]	[3401/3657]
**TaqMan**	OR (95% CIs)	1.03 (0.94–1.14)	1.12 (0.74–1.72)	1.00 (0.82–1.21)	1.01 (0.85–1.20)	1.12 (0.73–1.71)	1.03 (0.93–1.14)
	*P*_Association_	0.484	0.588	0.986	0.930	0.608	0.586
	Study	6	6	6	6	6	6
	[Case/control]	[4970/5321]	[4970/5321]	[4970/5321]	[4970/5321]	[4970/5321]	[4970/5321]
**Urinary system tumor**	OR (95% CIs)	1.04 (0.90–1.20)	1.01 (0.52–1.98)	1.00 (0.67–1.49)	1.00 (0.69–1.45)	1.00 (0.51–1.94)	1.04 (0.89–1.20)
	*P*_Association_	0.591	0.966	0.991	0.994	0.994	0.639
	Study	3	3	3	3	3	3
	[Case/control]	[2380/2519]	[2380/2519]	[2380/2519]	[2380/2519]	[2380/2519]	[2380/2519]
**Digestive system tumor**	OR (95% CIs)	1.05 (0.93–1.19)	1.02 (0.61–1.74)	1.06 (0.93–1.21)	1.06 (0.93–1.21)	1.02 (0.60–1.72)	1.05 (0.92–1.19)
	*P*_Association_	0.404	0.927	0.378	0.380	0.947	0.452
	Study	3	3	3	3	3	3
	[Case/control]	[3296/3369]	[3296/3369]	[3296/3369]	[3296/3369]	[3296/3369]	[3296/3369]

Abbreviations: CI, confidence interval; HB, hospital-based; OR, odds ratio; PB, population-based.

**Figure 4:**
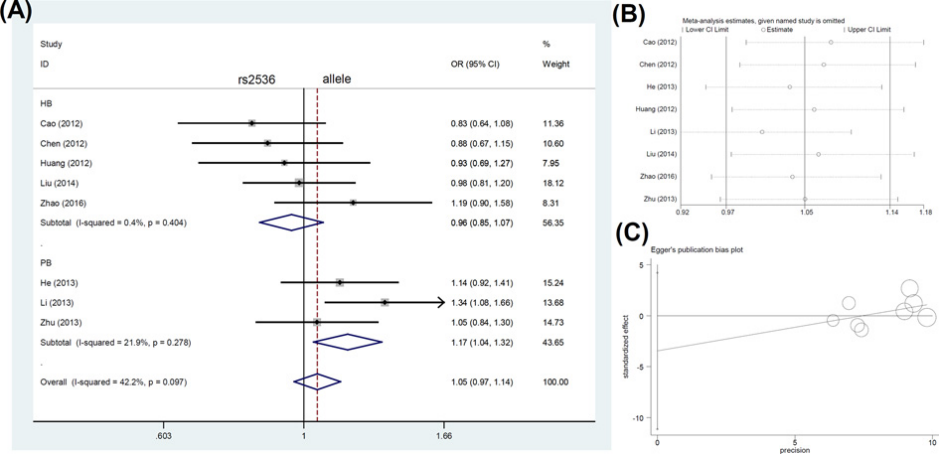
Pooling analysis of *mTOR* rs2536 under the allelic model (**A**) Forest plot of subgroup analysis by control source. (**B**) Begg’s test. (**C**) Sensitivity analysis data.

We present the forest plot data of the subgroup analysis by control source according to the genotype comparisons in Figure 4A (allele comparison), Supplementary Figure S7A (homozygote comparison), Supplementary Figure S8A (heterozygote comparison), Supplementary Figure S9A (dominant comparison), Supplementary Figure S10A (recessive comparison), and Supplementary Figure S11A (carrier comparison). We also provide the forest plot data for the subgroup analyses by genotyping method (Supplementary Figure S12) and cancer type (Supplementary Figure S13).

### Heterogeneity, publication bias, and sensitivity analysis

We used a random-effects model for the meta-analyses of rs2295080 via the allele, homozygote, heterozygote, dominant, and recessive genetic comparisons because substantial between-study heterogeneity was detected [Table 4, *I*^2^ value >50.0% or *P*_Heterogeneity_ <0.05]. For rs2536, a random-effects model was used in the heterozygote (Table 4, *I*^2^ value = 57.3%, *P*_Heterogeneity_= 0.022) and dominant (*I*^2^ value = 52.6%, *P*_Heterogeneity_= 0.039) comparisons.

**Table 4:** Heterogeneity and publication bias analysis of *mTOR* polymorphisms

SNP	Statistical analysis	Result	Allele	Homozygote	Heterozygote	Dominant	Recessive	Carrier
**rs2295080**	**Heterogeneity**	*I*^2^	69.2%	67.7%	48.5%	60.6%	64.8%	29.5%
		*P*_Heterogeneity_	<0.001	<0.001	0.016	0.001	<0.001	0.128
		Random/Fixed	Random	Random	Random	Random	Random	Fixed
	**Egger’s test**	*t*	1.02	1.09	−0.19	0.49	1.08	0.72
		*P*_Egger_	0.327	0.294	0.850	0.634	0.300	0.481
	**Begg’s test**	*z*	0.59	0.50	0.59	1.22	0.50	0.68
		*P*_Begg_	0.558	0.620	0.558	0.224	0.620	0.499
**rs2536**	**Heterogeneity**	*I*^2^	42.2%	0.0%	57.3%	52.6%	0.0%	32.4%
		*P*_Heterogeneity_	0.097	0.916	0.022	0.039	0.898	0.169
		Random/Fixed	Fixed	Fixed	Random	Random	Fixed	Fixed
	**Egger’s test**	*t*	−1.10	2.40	−1.58	−1.36	2.50	−1.32
		*P*_Egger_	0.313	0.053	0.166	0.223	0.046	0.235
	**Begg’s test**	*z*	0.37	2.10	0.62	0.62	1.86	0.62
		*P*_Begg_	0.711	0.035	0.536	0.536	0.063	0.536

SNP, single nucleotide polymorphism.

Our sensitivity analysis suggested the stability of the above data. The detailed plots are displayed in Figures 2B–4B, Supplementary Figures S1B–S4B, and Supplementary Figures S7B–S11B. In addition, we assessed publication bias through Egger’s and Begg’s tests. No large publication bias existed in the majority of genotype comparisons (Table 4, *P*_Egger_>0.05, *P*_Begg_>0.05), except for the homozygote (*P*_Begg_=0.035) and recessive (*P*_Egger_=0.046) comparisons of rs2536. The funnel plots of Egger’s test are presented in Figures 2C–4C, Supplementary Figures S1C–S4C, and Supplementary Figures S7C–S11C.

### FPRP analysis and TSA

**Figure 5:**
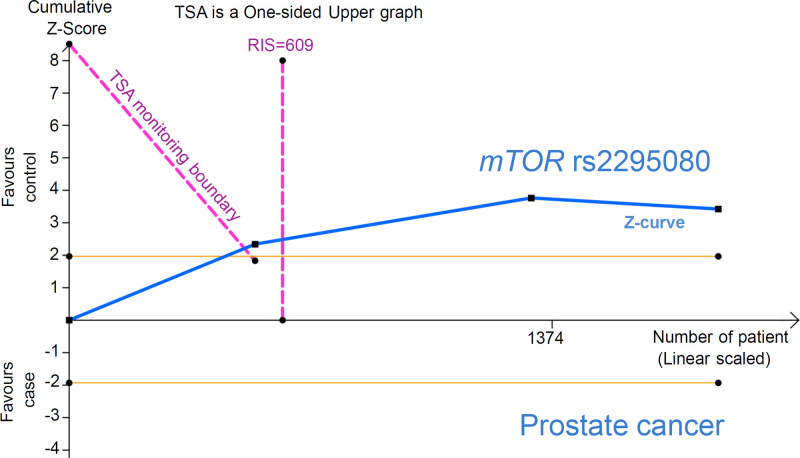
Trial sequential analysis for the association between *mTOR* rs2295080 and prostate cancer risk via the dominant comparison

To further minimize random errors to confirm the positive association between the *mTOR* rs2295080 polymorphism and the risk of urinary system tumors, prostate cancer, and leukemia, we performed FPRP analysis. As shown in Table 5, at a prior probability of 0.1, the FPRP values were all less than 0.2, and the statistical power values were larger than 0.99 for the allele, heterozygote, dominant and carrier comparisons in the assessment of urinary system tumor risk and for the heterozygote and dominant comparisons in the assessment of prostate cancer risk, suggesting a noteworthy association. TSA data for urinary system tumor risk (Supplementary Figure S14) further showed that the cumulative *Z*-curve crossed the TSA monitoring boundary and did not contact the RIS line, suggesting a robust conclusion, although the enrolled study number did not reach the required information size. With regard to the TSA data for prostate cancer risk (Figure 5), we observed that the *Z*-curve crossed both the TSA monitoring boundary and the RIS line, indicating a more robust conclusion.

**Table 5:** FPRP analysis for possible associations between the *mTOR* rs2295080 polymorphism and cancer risk

Subgroup	Model	OR (95% CI)	P^*^	Statistical Power	Prior probability level					
					0.25	0.1	0.01	0.001	0.0001	0.00001
Urinary system tumor	Allele	0.86 (0.76–0.98)	0.024	1.000	***0.066***	***0.175***	0.701	0.959	0.996	1.000
	Heterozygote	0.79 (0.71–0.88)	<0.001	0.999	***<0.001***	***<0.001***	***0.002***	***0.018***	***0.156***	0.649
	Dominant	0.80 (0.72–0.89)	<0.001	1.000	*<0.001*	***<0.001***	***0.004***	***0.039***	0.290	0.804
	Carrier	0.87 (0.79–0.96)	0.0056	1.000	***0.016***	***0.048***	*0.355*	*0.847*	*0.982*	0.998
Prostate cancer	Heterozygote	0.80 (0.70–0.90)	<0.001	0.999	***0.001***	***0.002***	*0.020*	*0.170*	0.672	0.953
	Dominant	0.82 (0.71–0.94)	0.004	0.999	***0.013***	***0.038***	0.304	0.815	0.978	0.998
	Carrier	0.88 (0.79–0.99)	0.033	1.000	***0.091***	0.231	0.768	0.971	0.997	1.000
leukemia	Allele	1.24 (1.05–1.47)	0.013	0.986	***0.039***	***0.108***	0.570	0.931	0.993	0.999
	Homozygote	2.25 (1.33–3.82)	0.003	0.067	***0.108***	0.265	0.799	0.976	0.998	1.000
	Recessive	2.25 (1.30–3.91)	0.004	0.075	***0.138***	0.325	0.841	0.982	0.998	1.000

Abbreviations: CI, 95% confidence interval; OR, odds ratio; *, *Chi-square test was used to calculate the genotype frequency distributions;* FPRP value < 0.2 in italics and bold.

We only observed that the FPRP value was less than 0.2 for the allele comparison in the assessment of leukemia, at a prior probability of 0.1 (Table 5). Furthermore, the cumulative *Z*-curve of leukemia risk did not exceed either the TSA monitoring boundary or the RIS line (Supplementary Figure S15), suggesting the need for more evidence for the association between *mTOR* rs2295080 and the risk of leukemia.

### eQTL analysis

**Figure 6:**
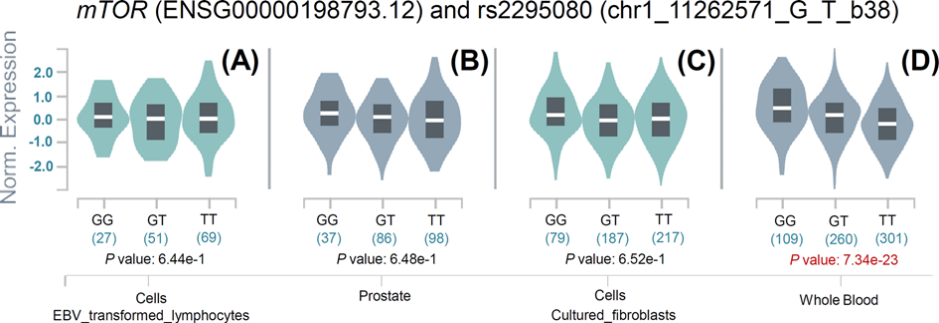
eQTL analysis of *mTOR* rs2295080 in certain cells or tissues within the GTEx database (**A**) Cells from the EBV_transformed_lymphocytes dataset; (**B**) prostate; (**C**) cells from the cultured_fibroblasts dataset; (**D**) whole blood.

Finally, we performed expression quantitative trait loci analysis of GTEx portal data to analyze the possible link between the rs2295080 (chr1_11262571_G_T_b38) and rs2536 (chr1_11106656_T_C_b38) SNPs and *mTOR* gene expression. As shown in Figure 6, we observed a potential correlation in whole blood samples (*P*=7.34e-23) but not in the prostate tissues or selected cells (EBV_transformed_lymphocytes and cultured_fibroblasts). With regard to rs2536, we did not observe a significant association between the SNPs and *mTOR* expression in most selected samples, except the cells in the cultured_fibroblasts dataset (Supplementary Figure S16, *P*=8.49e-4).

## Discussion

Publications with different conclusions on the effect of *mTOR* polymorphisms on cancer risk were retrieved. It was reported that *mTOR* rs2295080 may be associated with susceptibility to gastric cancer in the Chinese population [35,36]. However, a negative association between *mTOR* rs2295080 and the risk of gastric cancer in Chinese patients was also reported [40]. Therefore, the association between *mTOR* rs2295080 and overall cancer susceptibility has not been comprehensively evaluated. Different study enrolment and analysis strategies were applied in this study compared with four prior meta-analyses [13–16].

With regard to *mTOR* rs2295080, Zhu and colleagues conducted a meta-analysis of seven case–control studies and showed that *mTOR* rs2295080 may be associated with reduced cancer susceptibility in homozygous, heterozygous and dominant models [16]. In our study, we excluded one article [44] and added some new articles [29,33,35–38,40–42]. Because one article contained two case–control studies [41], nine new case-control studies were added in our meta-analysis of *mTOR* rs2295080.

In 2014, Shao et al. carried out a meta-analysis of *mTOR* rs2295080 containing five case–control studies [27,28,31,32,39] and reported a potential link between the wild-type TT genotype of the rs2295080 polymorphism and reduced cancer susceptibility under the dominant model [13]. Herein, we added 11 new case–control studies from 10 articles [16,29,33,35–38,40–42]. For the meta-analysis of rs2536, six case–control studies [27,28,30–32,43] were enrolled, and a negative association was detected via the dominant and recessive comparisons. In this study, we added two new case–control studies [34,42] for an updated meta-analysis.

In total, 10 case–control studies from 9 articles [16,27,28,31,32,36,38,39,41] were included in the meta-analysis of *mTOR* rs2295080 by Zining et al [15]. It was reported that the rs2295080 G allele was related to a reduced risk of genitourinary cancers under a dominant model and an increased risk of acute leukemia under a recessive model [15]. In addition, Zining et al conducted another meta-analysis of *mTOR* rs2536 containing seven case–control studies [27,28,30–32,43,45] and did not observe a positive association between *mTOR* rs2536 and cancer risk [15]. In the present study, we replaced one thesis [45] with another article with duplicate data [34] and added another new study [42].

Zhang and colleagues enrolled 10 case–control studies from nine articles [27,28,31,32,38,39,41,42,46] to conduct a meta-analysis of *mTOR* rs2295080 and performed subsequent subgroup analysis [14]. They observed a reduced susceptibility to urinary system tumors and digestive system tumors in the cases compared with the controls in GG vs. TT, TG vs. TT, GG+TG vs. TT, and GG vs. TG+TT comparisons (*P*<0.05, OR<1) [14], indicating the potential effect of the GG and TG genotypes of *mTOR* rs2295080 on the risk of urinary system tumors and digestive system tumors. However, an increased susceptibility to blood system tumors was observed only in the GG vs. TT comparison (*P*<0.05, OR>1). In the present study, we removed one study [46] and added eight new studies [12,16,29,33,35–37,40] to carry out an updated pooled analysis.

Our findings showed a reduced susceptibility to urinary system tumors in cases compared with controls via the allele (G vs. T), TG vs. TT, TG+GG vs. TT, and carrier (G vs. T) comparisons (*P*<0.05, OR<1) and a decreased risk of specific prostate cancers in cases compared with controls via the TG vs. TT, TG+GG vs. TT, and carrier (G vs. T) comparisons (*P*<0.05, OR<1). More importantly, we implemented FPRP analysis and TSA to confirm these associations. Nevertheless, we failed to detect a positive conclusion in the subgroups of studies related to digestive system tumors and specific gastric cancers. In addition, even though we also observed an increased risk of leukemia in cases compared with controls in the allele G vs. T, GG vs. TT, and GG vs. TT+TG comparisons (*P*<0.05, OR>1), the FPRP and TSA data suggested a lack of association.

Ten case-control studies from nine articles were enrolled in the meta-analysis of *mTOR* rs2536 by Zhang et al. and negative conclusions were observed in the overall meta-analysis and subsequent subgroup analyses [14]. In our study, two studies [45,46] with overlapping data were replaced with another two studies [27,34]. We thus included eight eligible case-control studies in the pooled analysis. We reached similar negative conclusions regarding the association between *mTOR* rs2536 and cancer risk in the overall population and in the subgroup of studies on “urinary system tumors” or “digestive system tumors”. Additionally, we added subgroup analyses based on “genotyping method” and “control source”. Although a negative result was detected in the subgroup of studies using “TaqMan” for genotyping and “HB” as the control source, there was a positive conclusion in the subgroup of studies using “PB” as the control source in the allele (G vs. A), AG vs. AA, AG+GG vs. AA, and carrier (G vs. A) comparisons (*P*<0.05, OR>1), suggesting a potential effect of the AG genotype of rs2536 on the susceptibility to cancer.

The following limitations should be noted. Owing to the very limited sample sizes, we failed to conduct subgroup analyses according to some specific cancer types, such as thyroid cancer and colorectal cancer. Additionally, all case–control studies were performed in the Chinese population. More data in the Caucasian population are needed. Several case–control studies did not utilize population-based controls. For example, we found that hospital-based controls were used in the subgroup of studies on “leukemia”. There was potential publication bias within the homozygote and recessive comparisons of rs2536. Genetic and environmental factors may contribute to this bias.

Taken together, our findings summarize currently published evidence comprehensive investigations regarding the genetic relationship between *mTOR* rs2295080/rs2536 polymorphisms and the risk of different cancers. We highlight the positive association between the TG genotype within the *mTOR* rs2295080 polymorphism and a reduced risk of urinary system tumors, especially prostate cancer, in the Chinese population. This will help researchers conduct further experiments to determine the molecular mechanisms. Considering the less than sufficient sample size for the pooled analysis of leukemia and the potential genetic relationship between *mTOR* gene expression and the rs2295080 polymorphism, relevant population-based clinical investigations by clinicians and researchers are warranted.

## Supplementary Material

Supplementary Figures S1-S16 and Tables S1-S2

## Abbreviations

*BRCA2*: BRCA2 DNA repair associated
CI: confidence interval
Embase: Excerpta Medica Database
eQTL: expression quantitative trait loci
FPRP: false-positive report probability
FRAP: FKBP12-rapamycin-associated protein
GTEx: The Genotype-Tissue Expression
HWE: Hardy–Weinberg equilibrium
mTOR: mammalian target of rapamycin
OR: odds ratio
PI3K: phosphatidylinositol 3-kinase
PRISMA: Preferred Reporting Items for Systematic Reviews and Meta-analyses
RIS: required information size
SNP: single-nucleotide polymorphism
TSA: trial sequential analysis
VEGF: vascular endothelial growth factor
