# Supplementary material for: Comprehensive analysis of the effect of rs2295080 and rs2536 polymorphisms within the mTOR gene on cancer risk
Source: Biosci Rep. 2020 Jul 9;40(7):BSR20191825. doi: 10.1042/BSR20191825 (PMC7350887; doi:10.1042/BSR20191825)
Supplement: Supplementary Figures S1-S16 and Tables S1-S2 [file BSR-2019-1825_supp.pdf]

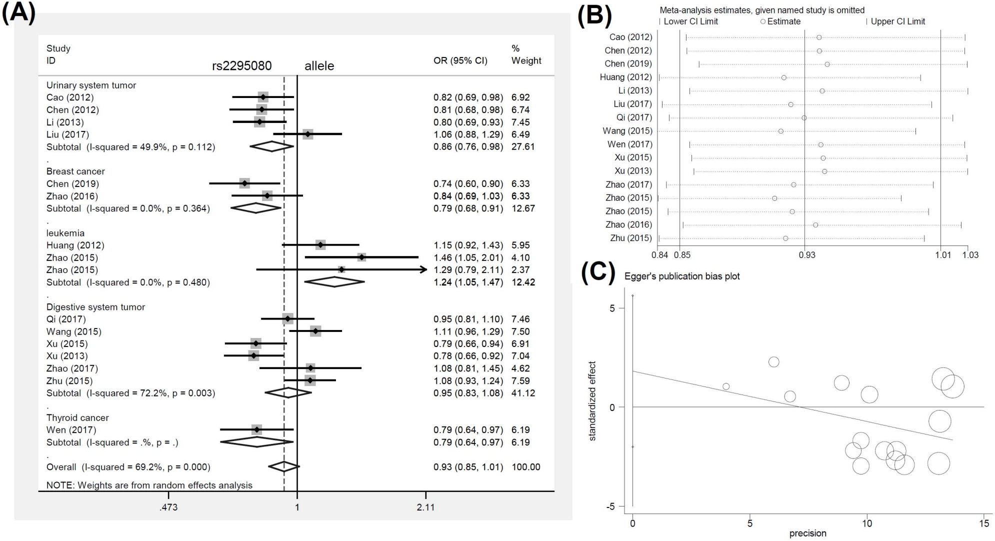
(A)
Study
ID
rs2295080 allele
OR (95% CI)
% Weight
Urinary system tumor
Cao (2012) 0.82 (0.69, 0.98) 6.92
Chen (2012) 0.81 (0.68, 0.98) 6.74
Li (2013) 0.80 (0.69, 0.93) 7.45
Liu (2017) 1.06 (0.88, 1.29) 6.49
Subtotal (I-squared = 49.9%, p = 0.112) 0.86 (0.76, 0.98) 27.61
Breast cancer
Chen (2019) 0.74 (0.60, 0.90) 6.33
Zhao (2016) 0.84 (0.69, 1.03) 6.33
Subtotal (I-squared = 0.0%, p = 0.364) 0.79 (0.68, 0.91) 12.67
leukemia
Huang (2012) 1.15 (0.92, 1.43) 5.95
Zhao (2015) 1.46 (1.05, 2.01) 4.10
Zhao (2015) 1.29 (0.79, 2.11) 2.37
Subtotal (I-squared = 0.0%, p = 0.480) 1.24 (1.05, 1.47) 12.42
Digestive system tumor
Qi (2017) 0.95 (0.81, 1.10) 7.46
Wang (2015) 1.11 (0.96, 1.29) 7.50
Xu (2015) 0.79 (0.66, 0.94) 6.91
Xu (2013) 0.78 (0.66, 0.92) 7.04
Zhao (2017) 1.08 (0.81, 1.45) 4.62
Zhu (2015) 1.08 (0.93, 1.24) 7.59
Subtotal (I-squared = 72.2%, p = 0.003) 0.95 (0.83, 1.08) 41.12
Thyroid cancer
Wen (2017) 0.79 (0.64, 0.97) 6.19
Subtotal (I-squared = .%, p = .) 0.79 (0.64, 0.97) 6.19
Overall (I-squared = 69.2%, p = 0.000) 0.93 (0.85, 1.01) 100.00
NOTE: Weights are from random effects analysis
.473
1
2.11
(B)
Meta-analysis estimates, given named study is omitted
Lower CI Limit
Estimate
Upper CI Limit
Cao (2012)
Chen (2012)
Chen (2019)
Huang (2012)
Li (2013)
Liu (2017)
Qi (2017)
Wang (2015)
Wen (2017)
Xu (2015)
Xu (2013)
Zhao (2017)
Zhao (2015)
Zhao (2015)
Zhao (2016)
Zhu (2015)
0.84 0.85
0.93
1.01 1.03
(C)
Egger's publication bias plot
standardized effect
precision
5
0
-5
0
5
10
15

Figure S1

Figure S1. **Pooled analysis of *mTOR* rs2295080 via the allele comparison. (**A**) Forest plot of subgroup analyses by cancer type. (**B) **Begg's test.** (C**) Sensitivity analysis.**

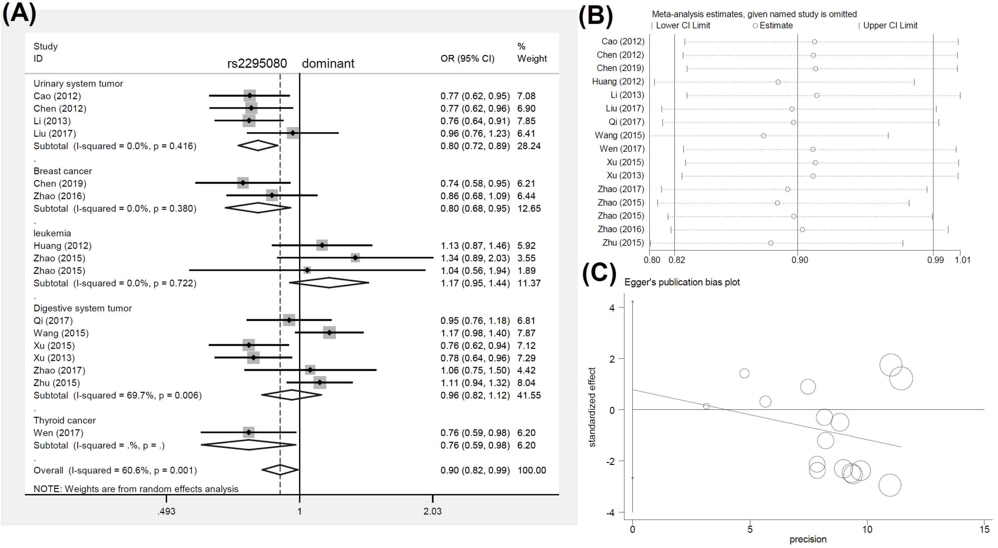
(A)
Study
ID
rs2295080 dominant
OR (95% CI)
% Weight
Urinary system tumor
Cao (2012) 0.77 (0.62, 0.95) 7.08
Chen (2012) 0.77 (0.62, 0.96) 6.90
Li (2013) 0.76 (0.64, 0.91) 7.85
Liu (2017) 0.96 (0.76, 1.23) 6.41
Subtotal (I-squared = 0.0%, p = 0.416) 0.80 (0.72, 0.89) 28.24
Breast cancer
Chen (2019) 0.74 (0.58, 0.95) 6.21
Zhao (2016) 0.86 (0.68, 1.09) 6.44
Subtotal (I-squared = 0.0%, p = 0.380) 0.80 (0.68, 0.95) 12.65
leukemia
Huang (2012) 1.13 (0.87, 1.46) 5.92
Zhao (2015) 1.34 (0.89, 2.03) 3.55
Zhao (2015) 1.04 (0.56, 1.94) 1.89
Subtotal (I-squared = 0.0%, p = 0.722) 1.17 (0.95, 1.44) 11.37
Digestive system tumor
Qi (2017) 0.95 (0.76, 1.18) 6.81
Wang (2015) 1.17 (0.98, 1.40) 7.87
Xu (2015) 0.76 (0.62, 0.94) 7.12
Xu (2013) 0.78 (0.64, 0.96) 7.29
Zhao (2017) 1.06 (0.75, 1.50) 4.42
Zhu (2015) 1.11 (0.94, 1.32) 8.04
Subtotal (I-squared = 69.7%, p = 0.006) 0.96 (0.82, 1.12) 41.55
Thyroid cancer
Wen (2017) 0.76 (0.59, 0.98) 6.20
Subtotal (I-squared = .%, p = .) 0.76 (0.59, 0.98) 6.20
Overall (I-squared = 60.6%, p = 0.001) 0.90 (0.82, 0.99) 100.00
NOTE: Weights are from random effects analysis
.493
1
2.03
(B)
Meta-analysis estimates, given named study is omitted
Lower CI Limit
Estimate
Upper CI Limit
Cao (2012)
Chen (2012)
Chen (2019)
Huang (2012)
Li (2013)
Liu (2017)
Qi (2017)
Wang (2015)
Wen (2017)
Xu (2015)
Xu (2013)
Zhao (2017)
Zhao (2015)
Zhao (2015)
Zhao (2016)
Zhu (2015)
0.80 0.82
0.90
0.99 1.01
(C)
Egger's publication bias plot
standardized effect
4
2
0
-2
-4
precision
0
5
10
15

Figure S2

Figure S2. **Pooled analysis of *mTOR* rs2295080 via the dominant comparison. (**A**) Forest plot of subgroup analysis by cancer type. (**B**) Begg's test. (**C**) Sensitivity analysis.**

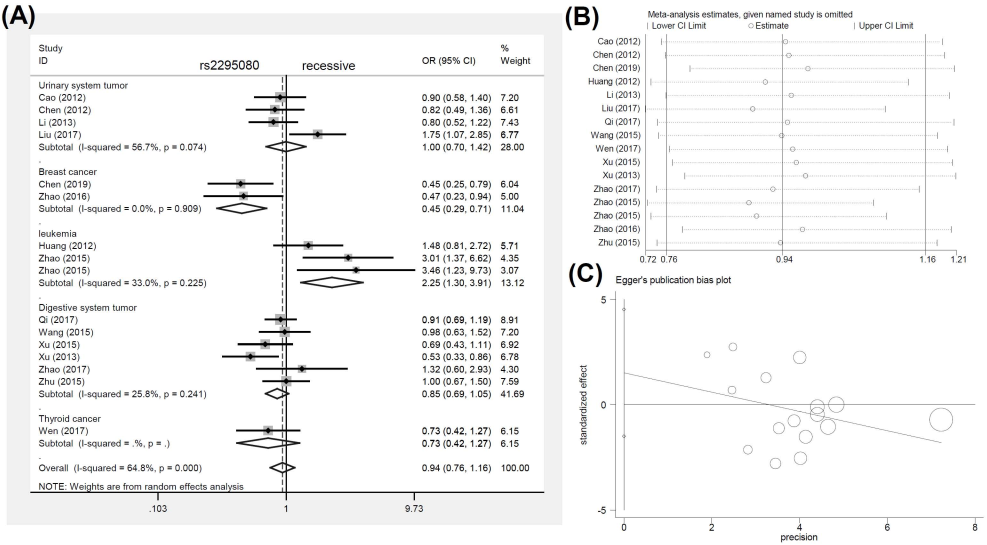

(A)
Study
ID
rs2295080
recessive
OR (95% CI)
%
Weight
Urinary system tumor
Cao (2012) 0.90 (0.58, 1.40) 7.20
Chen (2012) 0.82 (0.49, 1.36) 6.61
Li (2013) 0.80 (0.52, 1.22) 7.43
Liu (2017) 1.75 (1.07, 2.85) 6.77
Subtotal (I-squared = 56.7%, p = 0.074) 1.00 (0.70, 1.42) 28.00
Breast cancer
Chen (2019) 0.45 (0.25, 0.79) 6.04
Zhao (2016) 0.47 (0.23, 0.94) 5.00
Subtotal (I-squared = 0.0%, p = 0.909) 0.45 (0.29, 0.71) 11.04
leukemia
Huang (2012) 1.48 (0.81, 2.72) 5.71
Zhao (2015) 3.01 (1.37, 6.62) 4.35
Zhao (2015) 3.46 (1.23, 9.73) 3.07
Subtotal (I-squared = 33.0%, p = 0.225) 2.25 (1.30, 3.91) 13.12
Digestive system tumor
Qi (2017) 0.91 (0.69, 1.19) 8.91
Wang (2015) 0.98 (0.63, 1.52) 7.20
Xu (2015) 0.69 (0.43, 1.11) 6.92
Xu (2013) 0.53 (0.33, 0.86) 6.78
Zhao (2017) 1.32 (0.60, 2.93) 4.30
Zhu (2015) 1.00 (0.67, 1.50) 7.59
Subtotal (I-squared = 25.8%, p = 0.241) 0.85 (0.69, 1.05) 41.69
Thyroid cancer
Wen (2017) 0.73 (0.42, 1.27) 6.15
Subtotal (I-squared = .%, p = .) 0.73 (0.42, 1.27) 6.15
Overall (I-squared = 64.8%, p = 0.000) 0.94 (0.76, 1.16) 100.00
NOTE: Weights are from random effects analysis
.103
1
9.73
(B)
Meta-analysis estimates, given named study is omitted
Lower CI Limit
Estimate
Upper CI Limit
Cao (2012)
Chen (2012)
Chen (2019)
Huang (2012)
Li (2013)
Liu (2017)
Qi (2017)
Wang (2015)
Wen (2017)
Xu (2015)
Xu (2013)
Zhao (2017)
Zhao (2015)
Zhao (2015)
Zhao (2016)
Zhu (2015)
0.72 0.76
0.94
1.16 1.21
(C)
Egger's publication bias plot
standardized effect
precision
5
0
-5
0
2
4
6
8

Figure S3

Figure S3. **Pooled analysis of *mTOR* rs2295080 via the recessive comparison. (**A**) Forest plot of subgroup analysis by cancer type. (**B**) Begg's test. (**C**) Sensitivity analysis.**

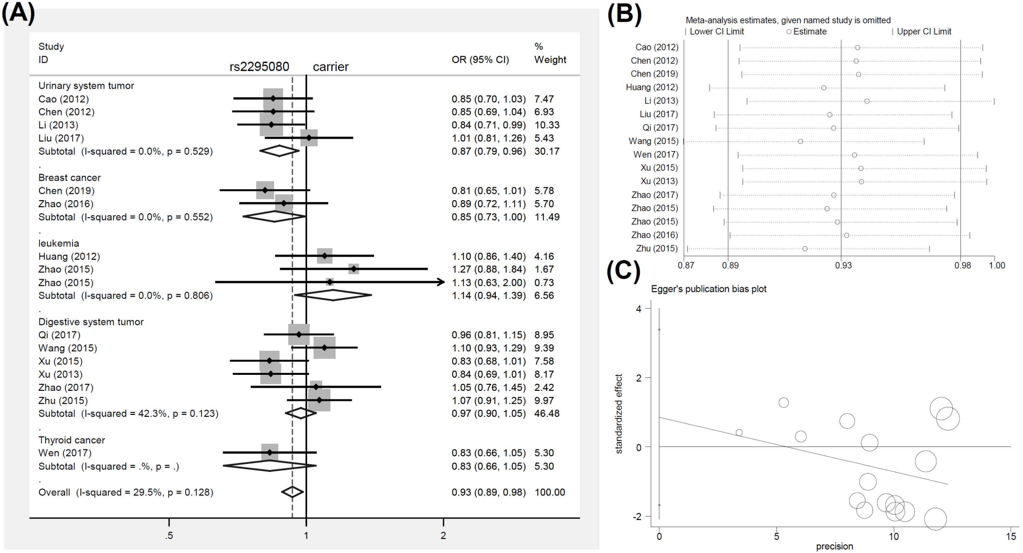
(A)
Study
ID
rs2295080 carrier
OR (95% CI)
% Weight
Urinary system tumor
Cao (2012) 0.85 (0.70, 1.03) 7.47
Chen (2012) 0.85 (0.69, 1.04) 6.93
Li (2013) 0.84 (0.71, 0.99) 10.33
Liu (2017) 1.01 (0.81, 1.26) 5.43
Subtotal (I-squared = 0.0%, p = 0.529) 0.87 (0.79, 0.96) 30.17
Breast cancer
Chen (2019) 0.81 (0.65, 1.01) 5.78
Zhao (2016) 0.89 (0.72, 1.11) 5.70
Subtotal (I-squared = 0.0%, p = 0.552) 0.85 (0.73, 1.00) 11.49
leukemia
Huang (2012) 1.10 (0.86, 1.40) 4.16
Zhao (2015) 1.27 (0.88, 1.84) 1.67
Zhao (2015) 1.13 (0.63, 2.00) 0.73
Subtotal (I-squared = 0.0%, p = 0.806) 1.14 (0.94, 1.39) 6.56
Digestive system tumor
Qi (2017) 0.96 (0.81, 1.15) 8.95
Wang (2015) 1.10 (0.93, 1.29) 9.39
Xu (2015) 0.83 (0.68, 1.01) 7.58
Xu (2013) 0.84 (0.69, 1.01) 8.17
Zhao (2017) 1.05 (0.76, 1.45) 2.42
Zhu (2015) 1.07 (0.91, 1.25) 9.97
Subtotal (I-squared = 42.3%, p = 0.123) 0.97 (0.90, 1.05) 46.48
Thyroid cancer
Wen (2017) 0.83 (0.66, 1.05) 5.30
Subtotal (I-squared = .%, p = .) 0.83 (0.66, 1.05) 5.30
Overall (I-squared = 29.5%, p = 0.128) 0.93 (0.89, 0.98) 100.00
.5
1
2
(B)
Meta-analysis estimates, given named study is omitted
Lower CI Limit
Estimate
Upper CI Limit
Cao (2012)
Chen (2012)
Chen (2019)
Huang (2012)
Li (2013)
Liu (2017)
Qi (2017)
Wang (2015)
Wen (2017)
Xu (2015)
Xu (2013)
Zhao (2017)
Zhao (2015)
Zhao (2015)
Zhao (2016)
Zhu (2015)
0.87
0.89
0.93
0.98
1.00
(C)
Egger's publication bias plot
standardized effect
precision
4
2
0
-2
0
5
10
15

Figure S4

Figure S4. **Pooled analysis of *mTOR* rs2295080 via the carrier comparison. (**A**) Forest plot of subgroup analysis by cancer type. (**B**) Begg's test. (**C**) Sensitivity analysis.**

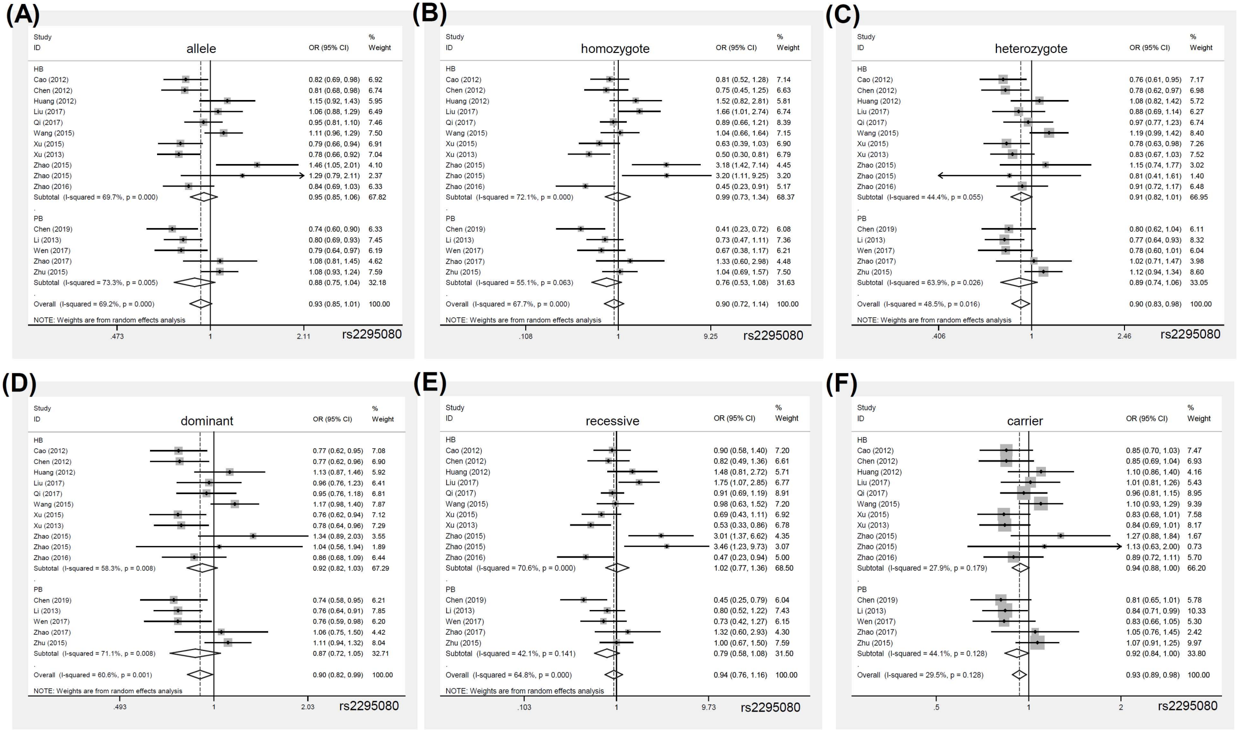
(A)
Study ID
allele
OR (95% CI)
% Weight
HB
Cao (2012) 0.82 (0.69, 0.98) 6.92
Chen (2012) 0.81 (0.68, 0.98) 6.74
Huang (2012) 1.15 (0.92, 1.43) 5.95
Liu (2017) 1.06 (0.88, 1.29) 6.49
Qi (2017) 0.95 (0.81, 1.10) 7.46
Wang (2015) 1.11 (0.96, 1.29) 7.50
Xu (2015) 0.79 (0.66, 0.94) 6.91
Xu (2013) 0.78 (0.66, 0.92) 7.04
Zhao (2015) 1.46 (1.05, 2.01) 4.10
Zhao (2015) 1.29 (0.79, 2.11) 2.37
Zhao (2016) 0.84 (0.69, 1.03) 6.33
Subtotal (I-squared = 69.7%, p = 0.000) 0.95 (0.85, 1.06) 67.82
PB
Chen (2019) 0.74 (0.60, 0.90) 6.33
Li (2013) 0.80 (0.69, 0.93) 7.45
Wen (2017) 0.79 (0.64, 0.97) 6.19
Zhao (2017) 1.08 (0.81, 1.45) 4.62
Zhu (2015) 1.08 (0.93, 1.24) 7.59
Subtotal (I-squared = 73.3%, p = 0.005) 0.88 (0.75, 1.04) 32.18
Overall (I-squared = 69.2%, p = 0.000) 0.93 (0.85, 1.01) 100.00
NOTE: Weights are from random effects analysis
.473
1
2.11
rs2295080
(B)
Study ID
homozygote
OR (95% CI)
% Weight
HB
Cao (2012) 0.81 (0.52, 1.28) 7.14
Chen (2012) 0.75 (0.45, 1.25) 6.63
Huang (2012) 1.52 (0.82, 2.81) 5.81
Liu (2017) 1.66 (1.01, 2.74) 6.74
Qi (2017) 0.89 (0.66, 1.21) 8.39
Wang (2015) 1.04 (0.66, 1.64) 7.15
Xu (2015) 0.63 (0.39, 1.03) 6.90
Xu (2013) 0.50 (0.30, 0.81) 6.79
Zhao (2015) 3.18 (1.42, 7.14) 4.45
Zhao (2015) 3.20 (1.11, 9.25) 3.20
Zhao (2016) 0.45 (0.23, 0.91) 5.17
Subtotal (I-squared = 72.1%, p = 0.000) 0.99 (0.73, 1.34) 68.37
PB
Chen (2019) 0.41 (0.23, 0.72) 6.08
Li (2013) 0.73 (0.47, 1.11) 7.36
Wen (2017) 0.67 (0.38, 1.17) 6.21
Zhao (2017) 1.33 (0.60, 2.98) 4.48
Zhu (2015) 1.04 (0.69, 1.57) 7.50
Subtotal (I-squared = 55.1%, p = 0.063) 0.76 (0.53, 1.08) 31.63
Overall (I-squared = 67.7%, p = 0.000) 0.90 (0.72, 1.14) 100.00
NOTE: Weights are from random effects analysis
.108
1
9.25
rs2295080
(C)
Study ID
heterozygote
OR (95% CI)
% Weight
HB
Cao (2012) 0.76 (0.61, 0.95) 7.17
Chen (2012) 0.78 (0.62, 0.97) 6.98
Huang (2012) 1.08 (0.82, 1.42) 5.72
Liu (2017) 0.88 (0.69, 1.14) 6.27
Qi (2017) 0.97 (0.77, 1.23) 6.74
Wang (2015) 1.19 (0.99, 1.42) 8.40
Xu (2015) 0.78 (0.63, 0.98) 7.26
Xu (2013) 0.83 (0.67, 1.03) 7.52
Zhao (2015) 1.15 (0.74, 1.77) 3.02
Zhao (2015) 0.81 (0.41, 1.61) 1.40
Zhao (2016) 0.91 (0.72, 1.17) 6.48
Subtotal (I-squared = 44.4%, p = 0.055) 0.91 (0.82, 1.01) 66.95
PB
Chen (2019) 0.80 (0.62, 1.04) 6.11
Li (2013) 0.77 (0.64, 0.93) 8.32
Wen (2017) 0.78 (0.60, 1.01) 6.04
Zhao (2017) 1.02 (0.71, 1.47) 3.98
Zhu (2015) 1.12 (0.94, 1.34) 8.60
Subtotal (I-squared = 63.9%, p = 0.026) 0.89 (0.74, 1.06) 33.05
Overall (I-squared = 48.5%, p = 0.016) 0.90 (0.83, 0.98) 100.00
NOTE: Weights are from random effects analysis
.406
1
2.46
rs2295080
(D)
Study ID
dominant
OR (95% CI)
% Weight
HB
Cao (2012) 0.77 (0.62, 0.95) 7.08
Chen (2012) 0.77 (0.62, 0.96) 6.90
Huang (2012) 1.13 (0.87, 1.46) 5.92
Liu (2017) 0.96 (0.76, 1.23) 6.41
Qi (2017) 0.95 (0.76, 1.18) 6.81
Wang (2015) 1.17 (0.98, 1.40) 7.87
Xu (2015) 0.76 (0.62, 0.94) 7.12
Xu (2013) 0.78 (0.64, 0.96) 7.29
Zhao (2015) 1.34 (0.89, 2.03) 3.55
Zhao (2015) 1.04 (0.56, 1.94) 1.89
Zhao (2016) 0.86 (0.68, 1.09) 6.44
Subtotal (I-squared = 58.3%, p = 0.008) 0.92 (0.82, 1.03) 67.29
PB
Chen (2019) 0.74 (0.58, 0.95) 6.21
Li (2013) 0.76 (0.64, 0.91) 7.85
Wen (2017) 0.76 (0.59, 0.98) 6.20
Zhao (2017) 1.06 (0.75, 1.50) 4.42
Zhu (2015) 1.11 (0.94, 1.32) 8.04
Subtotal (I-squared = 71.1%, p = 0.008) 0.87 (0.72, 1.05) 32.71
Overall (I-squared = 60.6%, p = 0.001) 0.90 (0.82, 0.99) 100.00
NOTE: Weights are from random effects analysis
.493
1
2.03
rs2295080
(E)
Study ID
recessive
OR (95% CI)
% Weight
HB
Cao (2012) 0.90 (0.58, 1.40) 7.20
Chen (2012) 0.82 (0.49, 1.36) 6.61
Huang (2012) 1.48 (0.81, 2.72) 5.71
Liu (2017) 1.75 (1.07, 2.85) 6.77
Qi (2017) 0.91 (0.69, 1.19) 8.91
Wang (2015) 0.98 (0.63, 1.52) 7.20
Xu (2015) 0.69 (0.43, 1.11) 6.92
Xu (2013) 0.53 (0.33, 0.86) 6.78
Zhao (2015) 3.01 (1.37, 6.62) 4.35
Zhao (2015) 3.46 (1.23, 9.73) 3.07
Zhao (2016) 0.47 (0.23, 0.94) 5.00
Subtotal (I-squared = 70.6%, p = 0.000) 1.02 (0.77, 1.36) 68.50
PB
Chen (2019) 0.45 (0.25, 0.79) 6.04
Li (2013) 0.80 (0.52, 1.22) 7.43
Wen (2017) 0.73 (0.42, 1.27) 6.15
Zhao (2017) 1.32 (0.60, 2.93) 4.30
Zhu (2015) 1.00 (0.67, 1.50) 7.59
Subtotal (I-squared = 42.1%, p = 0.141) 0.79 (0.58, 1.08) 31.50
Overall (I-squared = 64.8%, p = 0.000) 0.94 (0.76, 1.16) 100.00
NOTE: Weights are from random effects analysis
.103
1
9.73
rs2295080
(F)
Study ID
carrier
OR (95% CI)
% Weight
HB
Cao (2012) 0.85 (0.70, 1.03) 7.47
Chen (2012) 0.85 (0.69, 1.04) 6.93
Huang (2012) 1.10 (0.86, 1.40) 4.16
Liu (2017) 1.01 (0.81, 1.26) 5.43
Qi (2017) 0.96 (0.81, 1.15) 8.95
Wang (2015) 1.10 (0.93, 1.29) 9.39
Xu (2015) 0.83 (0.68, 1.01) 7.58
Xu (2013) 0.84 (0.69, 1.01) 8.17
Zhao (2015) 1.27 (0.88, 1.84) 1.67
Zhao (2015) 1.13 (0.63, 2.00) 0.73
Zhao (2016) 0.89 (0.72, 1.11) 5.70
Subtotal (I-squared = 27.9%, p = 0.179) 0.94 (0.88, 1.00) 66.20
PB
Chen (2019) 0.81 (0.65, 1.01) 5.78
Li (2013) 0.84 (0.71, 0.99) 10.33
Wen (2017) 0.83 (0.66, 1.05) 5.30
Zhao (2017) 1.05 (0.76, 1.45) 2.42
Zhu (2015) 1.07 (0.91, 1.25) 9.97
Subtotal (I-squared = 44.1%, p = 0.128) 0.92 (0.84, 1.00) 33.80
Overall (I-squared = 29.5%, p = 0.128) 0.93 (0.89, 0.98) 100.00
.5
1
2
rs2295080

Figure S5

Figure S5. **Forest plot of subgroup analyses of *mTOR* rs2295080 by control source. (**A**) Allele; (**B**) homozygote; (**C**) heterozygote; (**D**) dominant; (**E**) recessive; (**F**) carrier.**

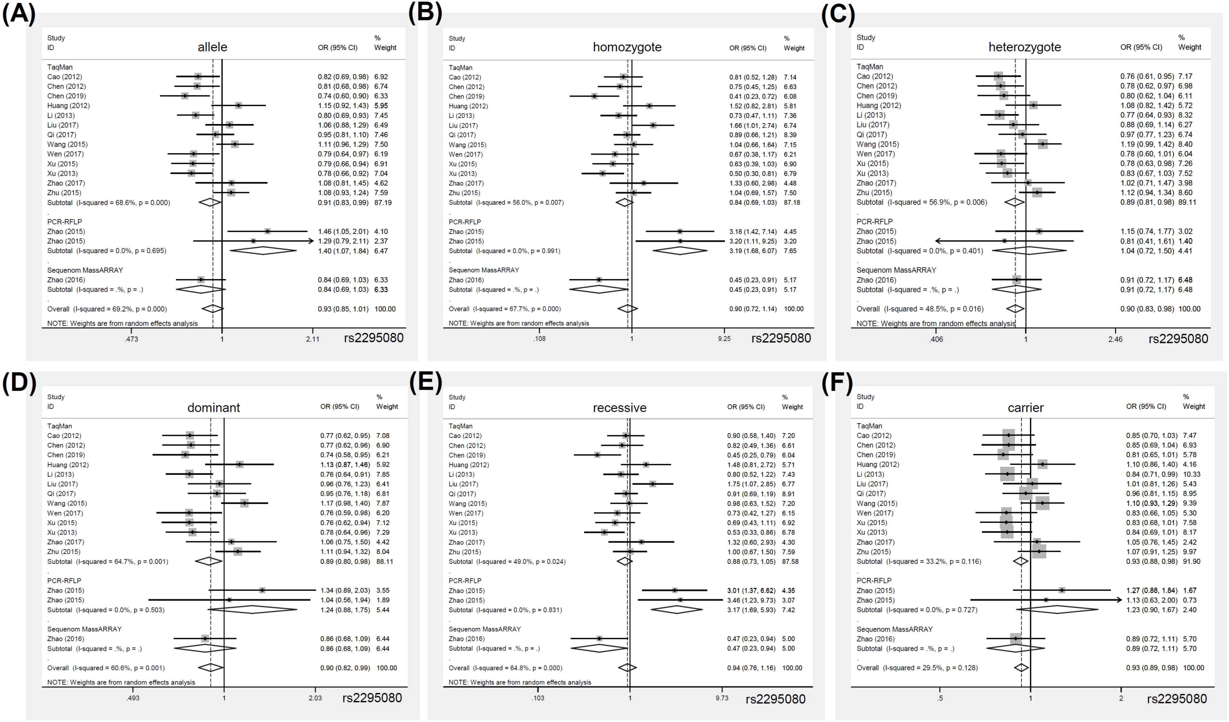
(A)
Study ID
allele
OR (95% CI)
% Weight
TaqMan
Cao (2012) 0.82 (0.69, 0.98) 6.92
Chen (2012) 0.81 (0.68, 0.98) 6.74
Chen (2019) 0.74 (0.60, 0.90) 6.33
Huang (2012) 1.15 (0.92, 1.43) 5.95
Li (2013) 0.80 (0.69, 0.93) 7.45
Liu (2017) 1.06 (0.88, 1.29) 6.49
Qi (2017) 0.95 (0.81, 1.10) 7.46
Wang (2015) 1.11 (0.96, 1.29) 7.50
Wen (2017) 0.79 (0.64, 0.97) 6.19
Xu (2015) 0.79 (0.66, 0.94) 6.91
Xu (2013) 0.78 (0.66, 0.92) 7.04
Zhao (2017) 1.08 (0.81, 1.45) 4.62
Zhu (2015) 1.08 (0.93, 1.24) 7.59
Subtotal (I-squared = 68.6%, p = 0.000) 0.91 (0.83, 0.99) 87.19
PCR-RFLP
Zhao (2015) 1.46 (1.05, 2.01) 4.10
Zhao (2015) 1.29 (0.79, 2.11) 2.37
Subtotal (I-squared = 0.0%, p = 0.695) 1.40 (1.07, 1.84) 6.47
Sequenom MassARRAY
Zhao (2016) 0.84 (0.69, 1.03) 6.33
Subtotal (I-squared = .%, p = .) 0.84 (0.69, 1.03) 6.33
Overall (I-squared = 69.2%, p = 0.000) 0.93 (0.85, 1.01) 100.00
NOTE: Weights are from random effects analysis
.473
1
2.11
rs2295080
(B)
Study ID
homozygote
OR (95% CI)
% Weight
TaqMan
Cao (2012) 0.81 (0.52, 1.28) 7.14
Chen (2012) 0.75 (0.45, 1.25) 6.63
Chen (2019) 0.41 (0.23, 0.72) 6.08
Huang (2012) 1.52 (0.82, 2.81) 5.81
Li (2013) 0.73 (0.47, 1.11) 7.36
Liu (2017) 1.66 (1.01, 2.74) 6.74
Qi (2017) 0.89 (0.66, 1.21) 8.39
Wang (2015) 1.04 (0.66, 1.64) 7.15
Wen (2017) 0.67 (0.38, 1.17) 6.21
Xu (2015) 0.63 (0.39, 1.03) 6.90
Xu (2013) 0.50 (0.30, 0.81) 6.79
Zhao (2017) 1.33 (0.60, 2.98) 4.48
Zhu (2015) 1.04 (0.69, 1.57) 7.50
Subtotal (I-squared = 56.0%, p = 0.007) 0.84 (0.69, 1.03) 87.18
PCR-RFLP
Zhao (2015) 3.18 (1.42, 7.14) 4.45
Zhao (2015) 3.20 (1.11, 9.25) 3.20
Subtotal (I-squared = 0.0%, p = 0.991) 3.19 (1.68, 6.07) 7.65
Sequenom MassARRAY
Zhao (2016) 0.45 (0.23, 0.91) 5.17
Subtotal (I-squared = .%, p = .) 0.45 (0.23, 0.91) 5.17
Overall (I-squared = 67.7%, p = 0.000) 0.90 (0.72, 1.14) 100.00
NOTE: Weights are from random effects analysis
.108
1
9.25
rs2295080
(C)
Study ID
heterozygote
OR (95% CI)
% Weight
TaqMan
Cao (2012) 0.76 (0.61, 0.95) 7.17
Chen (2012) 0.78 (0.62, 0.97) 6.98
Chen (2019) 0.80 (0.62, 1.04) 6.11
Huang (2012) 1.08 (0.82, 1.42) 5.72
Li (2013) 0.77 (0.64, 0.93) 8.32
Liu (2017) 0.88 (0.69, 1.14) 6.27
Qi (2017) 0.97 (0.77, 1.23) 6.74
Wang (2015) 1.19 (0.99, 1.42) 8.40
Wen (2017) 0.78 (0.60, 1.01) 6.04
Xu (2015) 0.78 (0.63, 0.98) 7.26
Xu (2013) 0.83 (0.67, 1.03) 7.52
Zhao (2017) 1.02 (0.71, 1.47) 3.98
Zhu (2015) 1.12 (0.94, 1.34) 8.60
Subtotal (I-squared = 56.9%, p = 0.006) 0.89 (0.81, 0.98) 89.11
PCR-RFLP
Zhao (2015) 1.15 (0.74, 1.77) 3.02
Zhao (2015) 0.81 (0.41, 1.61) 1.40
Subtotal (I-squared = 0.0%, p = 0.401) 1.04 (0.72, 1.50) 4.41
Sequenom MassARRAY
Zhao (2016) 0.91 (0.72, 1.17) 6.48
Subtotal (I-squared = .%, p = .) 0.91 (0.72, 1.17) 6.48
Overall (I-squared = 48.5%, p = 0.016) 0.90 (0.83, 0.98) 100.00
NOTE: Weights are from random effects analysis
.406
1
2.46
rs2295080
(D)
Study ID
dominant
OR (95% CI)
% Weight
TaqMan
Cao (2012) 0.77 (0.62, 0.95) 7.08
Chen (2012) 0.77 (0.62, 0.96) 6.90
Chen (2019) 0.74 (0.58, 0.95) 6.21
Huang (2012) 1.13 (0.87, 1.46) 5.92
Li (2013) 0.76 (0.64, 0.91) 7.85
Liu (2017) 0.96 (0.76, 1.23) 6.41
Qi (2017) 0.95 (0.76, 1.18) 6.81
Wang (2015) 1.17 (0.98, 1.40) 7.87
Wen (2017) 0.76 (0.59, 0.98) 6.20
Xu (2015) 0.76 (0.62, 0.94) 7.12
Xu (2013) 0.78 (0.64, 0.96) 7.29
Zhao (2017) 1.06 (0.75, 1.50) 4.42
Zhu (2015) 1.11 (0.94, 1.32) 8.04
Subtotal (I-squared = 64.7%, p = 0.001) 0.89 (0.80, 0.98) 88.11
PCR-RFLP
Zhao (2015) 1.34 (0.89, 2.03) 3.55
Zhao (2015) 1.04 (0.56, 1.94) 1.89
Subtotal (I-squared = 0.0%, p = 0.503) 1.24 (0.88, 1.75) 5.44
Sequenom MassARRAY
Zhao (2016) 0.86 (0.68, 1.09) 6.44
Subtotal (I-squared = .%, p = .) 0.86 (0.68, 1.09) 6.44
Overall (I-squared = 60.6%, p = 0.001) 0.90 (0.82, 0.99) 100.00
NOTE: Weights are from random effects analysis
.493
1
2.03
rs2295080
(E)
Study ID
recessive
OR (95% CI)
% Weight
TaqMan
Cao (2012) 0.90 (0.58, 1.40) 7.20
Chen (2012) 0.82 (0.49, 1.36) 6.61
Chen (2019) 0.45 (0.25, 0.79) 6.04
Huang (2012) 1.48 (0.81, 2.72) 5.71
Li (2013) 0.80 (0.52, 1.22) 7.43
Liu (2017) 1.75 (1.07, 2.85) 6.77
Qi (2017) 0.91 (0.69, 1.19) 8.91
Wang (2015) 0.98 (0.63, 1.52) 7.20
Wen (2017) 0.73 (0.42, 1.27) 6.15
Xu (2015) 0.69 (0.43, 1.11) 6.92
Xu (2013) 0.53 (0.33, 0.86) 6.78
Zhao (2017) 1.32 (0.60, 2.93) 4.30
Zhu (2015) 1.00 (0.67, 1.50) 7.59
Subtotal (I-squared = 49.0%, p = 0.024) 0.88 (0.73, 1.05) 87.58
PCR-RFLP
Zhao (2015) 3.01 (1.37, 6.62) 4.35
Zhao (2015) 3.46 (1.23, 9.73) 3.07
Subtotal (I-squared = 0.0%, p = 0.831) 3.17 (1.69, 5.93) 7.42
Sequenom MassARRAY
Zhao (2016) 0.47 (0.23, 0.94) 5.00
Subtotal (I-squared = .%, p = .) 0.47 (0.23, 0.94) 5.00
Overall (I-squared = 64.8%, p = 0.000) 0.94 (0.76, 1.16) 100.00
NOTE: Weights are from random effects analysis
.103
1
9.73
rs2295080
(F)
Study ID
carrier
OR (95% CI)
% Weight
TaqMan
Cao (2012) 0.85 (0.70, 1.03) 7.47
Chen (2012) 0.85 (0.69, 1.04) 6.93
Chen (2019) 0.81 (0.65, 1.01) 5.78
Huang (2012) 1.10 (0.86, 1.40) 4.16
Li (2013) 0.84 (0.71, 0.99) 10.33
Liu (2017) 1.01 (0.81, 1.26) 5.43
Qi (2017) 0.96 (0.81, 1.15) 8.95
Wang (2015) 1.10 (0.93, 1.29) 9.39
Wen (2017) 0.83 (0.66, 1.05) 5.30
Xu (2015) 0.83 (0.68, 1.01) 7.58
Xu (2013) 0.84 (0.69, 1.01) 8.17
Zhao (2017) 1.05 (0.76, 1.45) 2.42
Zhu (2015) 1.07 (0.91, 1.25) 9.97
Subtotal (I-squared = 33.2%, p = 0.116) 0.93 (0.88, 0.98) 91.90
PCR-RFLP
Zhao (2015) 1.27 (0.88, 1.84) 1.67
Zhao (2015) 1.13 (0.63, 2.00) 0.73
Subtotal (I-squared = 0.0%, p = 0.727) 1.23 (0.90, 1.67) 2.40
Sequenom MassARRAY
Zhao (2016) 0.89 (0.72, 1.11) 5.70
Subtotal (I-squared = .%, p = .) 0.89 (0.72, 1.11) 5.70
Overall (I-squared = 29.5%, p = 0.128) 0.93 (0.89, 0.98) 100.00
.5
1
2
rs2295080

Figure S6

Figure S6. **Forest plot of subgroup analyses of *mTOR* rs2295080 by genotyping method. (**A**) Allele; (**B**) homozygote; (**C**) heterozygote; (**D**) dominant; (**E**) recessive; (**F**) carrier.**

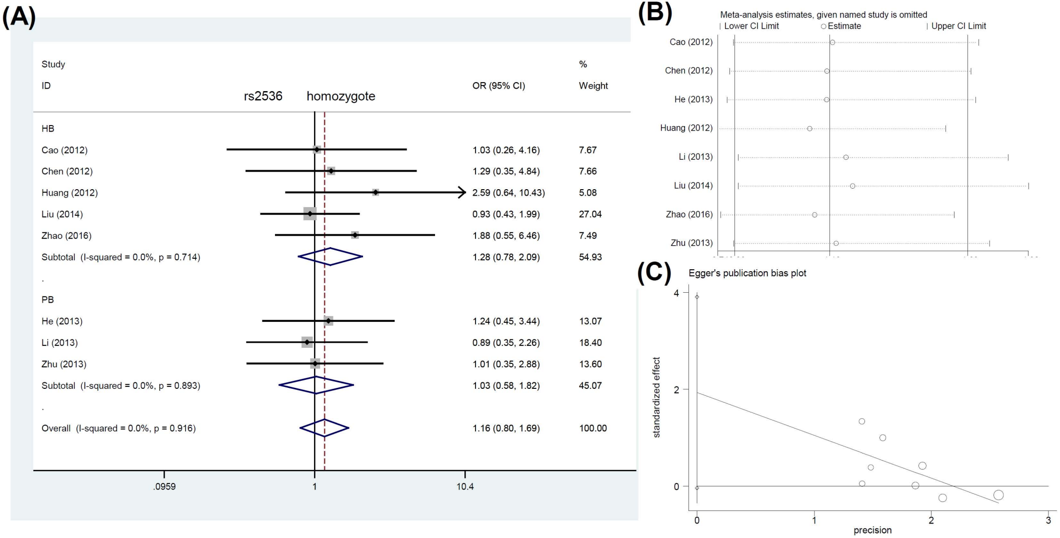
(A)
Study
ID
rs2536 homozygote
OR (95% CI)
% Weight
HB
Cao (2012) 1.03 (0.26, 4.16) 7.67
Chen (2012) 1.29 (0.35, 4.84) 7.66
Huang (2012) 2.59 (0.64, 10.43) 5.08
Liu (2014) 0.93 (0.43, 1.99) 27.04
Zhao (2016) 1.88 (0.55, 6.46) 7.49
Subtotal (I-squared = 0.0%, p = 0.714) 1.28 (0.78, 2.09) 54.93
PB
He (2013) 1.24 (0.45, 3.44) 13.07
Li (2013) 0.89 (0.35, 2.26) 18.40
Zhu (2013) 1.01 (0.35, 2.88) 13.60
Subtotal (I-squared = 0.0%, p = 0.893) 1.03 (0.58, 1.82) 45.07
Overall (I-squared = 0.0%, p = 0.916) 1.16 (0.80, 1.69) 100.00
.0959
1
10.4
(B)
Meta-analysis estimates, given named study is omitted
Lower CI Limit
Estimate
Upper CI Limit
Cao (2012)
Chen (2012)
He (2013)
Huang (2012)
Li (2013)
Liu (2014)
Zhao (2016)
Zhu (2013)
(C)
Egger's publication bias plot
standardized effect
precision
4
2
0
0
1
2
3

Figure S7

Figure S7. **Pooled analysis of *mTOR* rs2536 via the homozygote comparison. (**A**) Forest plot of subgroup analyses by control source. (**B**) Begg's test. (**C**) Sensitivity analysis.**

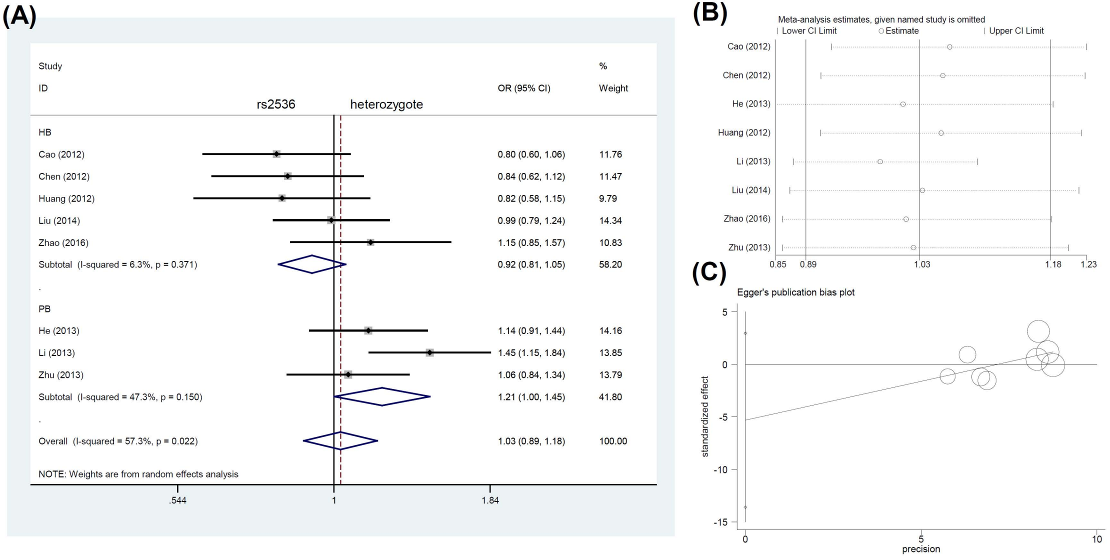
(A)
Study
ID
OR (95% CI)
%
Weight
rs2536
heterozygote
HB
Cao (2012) 0.80 (0.60, 1.06) 11.76
Chen (2012) 0.84 (0.62, 1.12) 11.47
Huang (2012) 0.82 (0.58, 1.15) 9.79
Liu (2014) 0.99 (0.79, 1.24) 14.34
Zhao (2016) 1.15 (0.85, 1.57) 10.83
Subtotal (I-squared = 6.3%, p = 0.371) 0.92 (0.81, 1.05) 58.20
PB
He (2013) 1.14 (0.91, 1.44) 14.16
Li (2013) 1.45 (1.15, 1.84) 13.85
Zhu (2013) 1.06 (0.84, 1.34) 13.79
Subtotal (I-squared = 47.3%, p = 0.150) 1.21 (1.00, 1.45) 41.80
Overall (I-squared = 57.3%, p = 0.022) 1.03 (0.89, 1.18) 100.00
NOTE: Weights are from random effects analysis
.544
1
1.84
(B)
Meta-analysis estimates, given named study is omitted
Lower CI Limit
Estimate
Upper CI Limit
Cao (2012)
Chen (2012)
He (2013)
Huang (2012)
Li (2013)
Liu (2014)
Zhao (2016)
Zhu (2013)
0.85
0.89
1.03
1.18
1.23
(C)
Egger's publication bias plot
standardized effect
precision
5
0
-5
-10
-15
0
5
10

Figure S8

Figure S8. **Pooled analysis of *mTOR* rs2536 via the heterozygote comparison. (**A**) Forest plot of subgroup analysis by control source. (**B**) Begg's test. (**C**) Sensitivity analysis.**

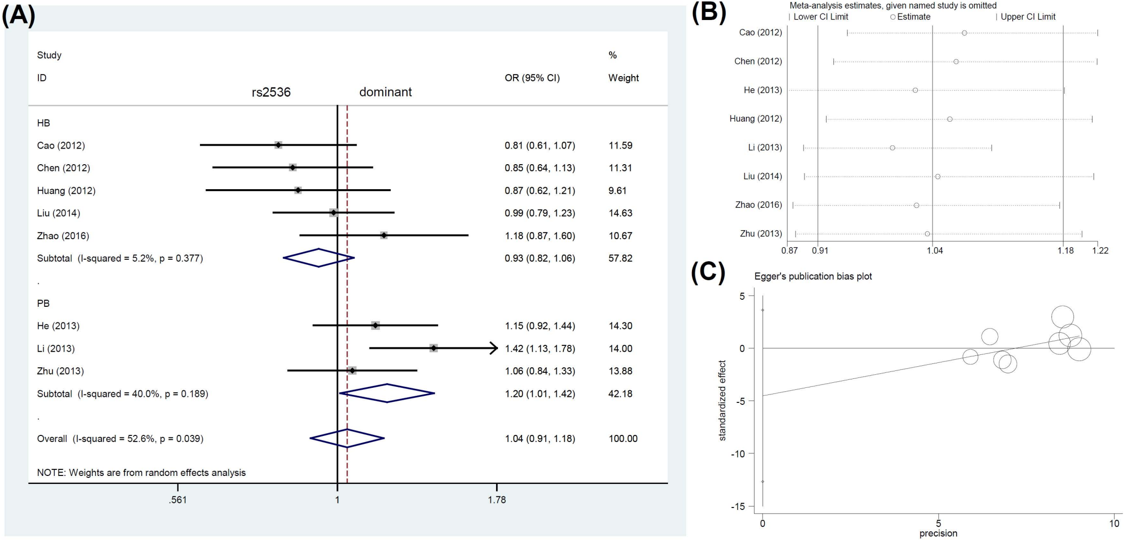

(A)
Study
ID
rs2536 dominant
OR (95% CI)
%
Weight
HB
Cao (2012) 0.81 (0.61, 1.07) 11.59
Chen (2012) 0.85 (0.64, 1.13) 11.31
Huang (2012) 0.87 (0.62, 1.21) 9.61
Liu (2014) 0.99 (0.79, 1.23) 14.63
Zhao (2016) 1.18 (0.87, 1.60) 10.67
Subtotal (I-squared = 5.2%, p = 0.377) 0.93 (0.82, 1.06) 57.82
PB
He (2013) 1.15 (0.92, 1.44) 14.30
Li (2013) 1.42 (1.13, 1.78) 14.00
Zhu (2013) 1.06 (0.84, 1.33) 13.88
Subtotal (I-squared = 40.0%, p = 0.189) 1.20 (1.01, 1.42) 42.18
Overall (I-squared = 52.6%, p = 0.039) 1.04 (0.91, 1.18) 100.00
NOTE: Weights are from random effects analysis
.561
1
1.78
(B)
Meta-analysis estimates, given named study is omitted
Lower CI Limit
Estimate
Upper CI Limit
Cao (2012)
Chen (2012)
He (2013)
Huang (2012)
Li (2013)
Liu (2014)
Zhao (2016)
Zhu (2013)
0.87
0.91
1.04
1.18
1.22
(C)
Egger's publication bias plot
standardized effect
5
0
-5
-10
-15
precision
0
5
10

Figure S9

Figure S9. **Pooled analysis of *mTOR* rs2536 via the dominant comparison. (**A**) Forest plot of subgroup analysis by control source. (**B**) Begg's test. (**C**) Sensitivity analysis.**

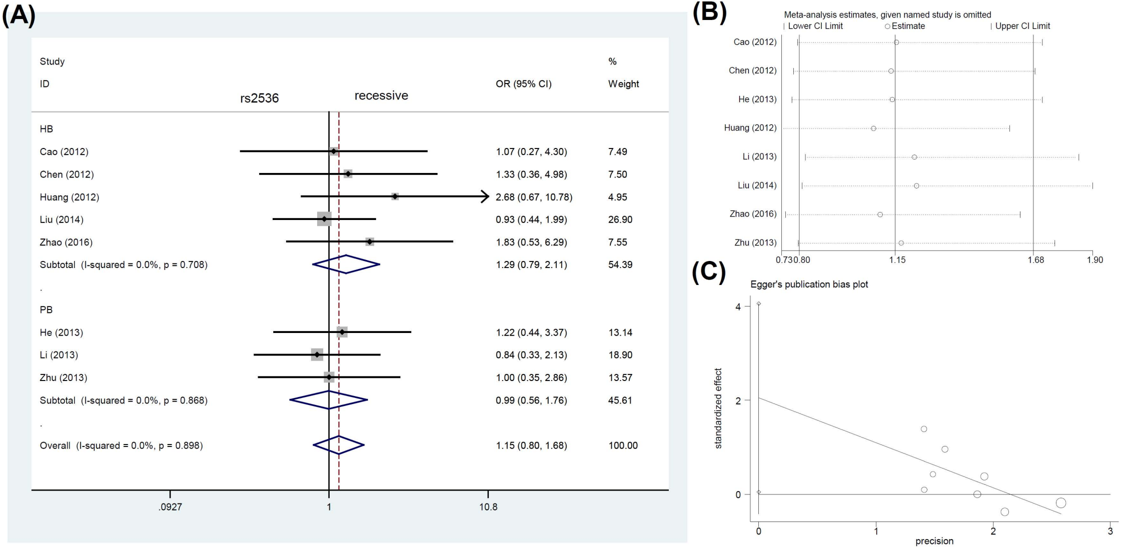
(A)
Study
ID
rs2536
recessive
OR (95% CI)
%
Weight
HB
Cao (2012) 1.07 (0.27, 4.30) 7.49
Chen (2012) 1.33 (0.36, 4.98) 7.50
Huang (2012) 2.68 (0.67, 10.78) 4.95
Liu (2014) 0.93 (0.44, 1.99) 26.90
Zhao (2016) 1.83 (0.53, 6.29) 7.55
Subtotal (I-squared = 0.0%, p = 0.708) 1.29 (0.79, 2.11) 54.39
PB
He (2013) 1.22 (0.44, 3.37) 13.14
Li (2013) 0.84 (0.33, 2.13) 18.90
Zhu (2013) 1.00 (0.35, 2.86) 13.57
Subtotal (I-squared = 0.0%, p = 0.868) 0.99 (0.56, 1.76) 45.61
Overall (I-squared = 0.0%, p = 0.898) 1.15 (0.80, 1.68) 100.00
.0927
1
10.8
(B)
Meta-analysis estimates, given named study is omitted
Lower CI Limit
Estimate
Upper CI Limit
Cao (2012)
Chen (2012)
He (2013)
Huang (2012)
Li (2013)
Liu (2014)
Zhao (2016)
Zhu (2013)
0.73
0.80
1.15
1.68
1.90
(C)
Egger's publication bias plot
standardized effect
precision
0
1
2
3
4

Figure S10

Figure S10. **Pooled analysis of *mTOR* rs2536 via the recessive comparison. (**A**) Forest plot of subgroup analysis by control source. (**B**) Begg's test. (**C**) Sensitivity analysis.**

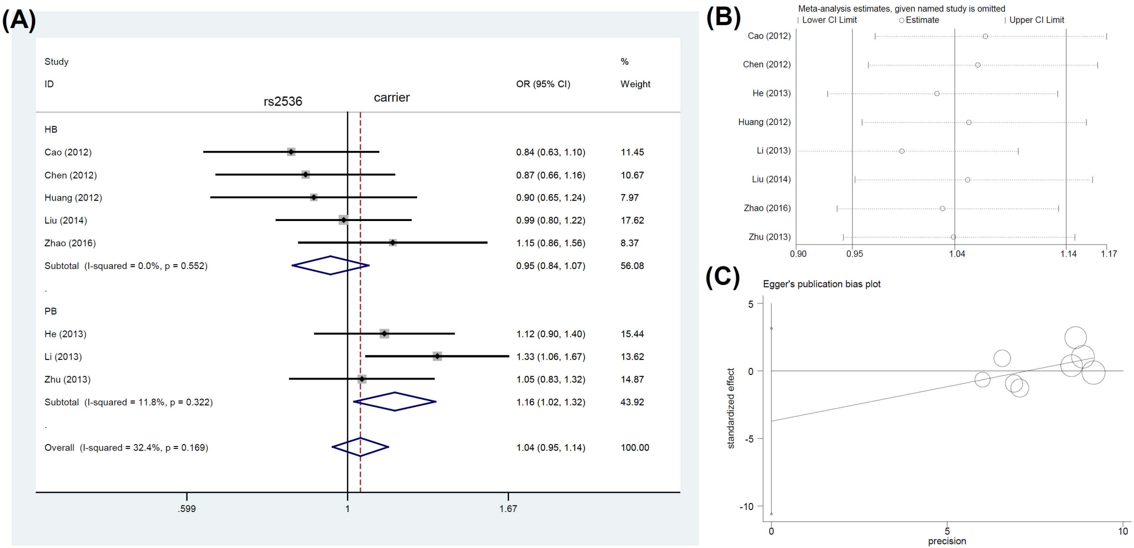
(A)
Study
ID
rs2536
carrier
OR (95% CI)
% Weight
HB
Cao (2012) 0.84 (0.63, 1.10) 11.45
Chen (2012) 0.87 (0.66, 1.16) 10.67
Huang (2012) 0.90 (0.65, 1.24) 7.97
Liu (2014) 0.99 (0.80, 1.22) 17.62
Zhao (2016) 1.15 (0.86, 1.56) 8.37
Subtotal (I-squared = 0.0%, p = 0.552) 0.95 (0.84, 1.07) 56.08
PB
He (2013) 1.12 (0.90, 1.40) 15.44
Li (2013) 1.33 (1.06, 1.67) 13.62
Zhu (2013) 1.05 (0.83, 1.32) 14.87
Subtotal (I-squared = 11.8%, p = 0.322) 1.16 (1.02, 1.32) 43.92
Overall (I-squared = 32.4%, p = 0.169) 1.04 (0.95, 1.14) 100.00
.599
1
1.67
(B)
Meta-analysis estimates, given named study is omitted
Lower CI Limit
Estimate
Upper CI Limit
Cao (2012)
Chen (2012)
He (2013)
Huang (2012)
Li (2013)
Liu (2014)
Zhao (2016)
Zhu (2013)
0.90
0.95
1.04
1.14
1.17
(C)
Egger's publication bias plot
standardized effect
5
0
-5
-10
precision
0
5
10

Figure S11

Figure S11. **Pooled analysis of *mTOR* rs2536 via the carrier comparison. (**A**) Forest plot of subgroup analysis by control source. (**B**) Begg's test.** (C**) Sensitivity analysis.**

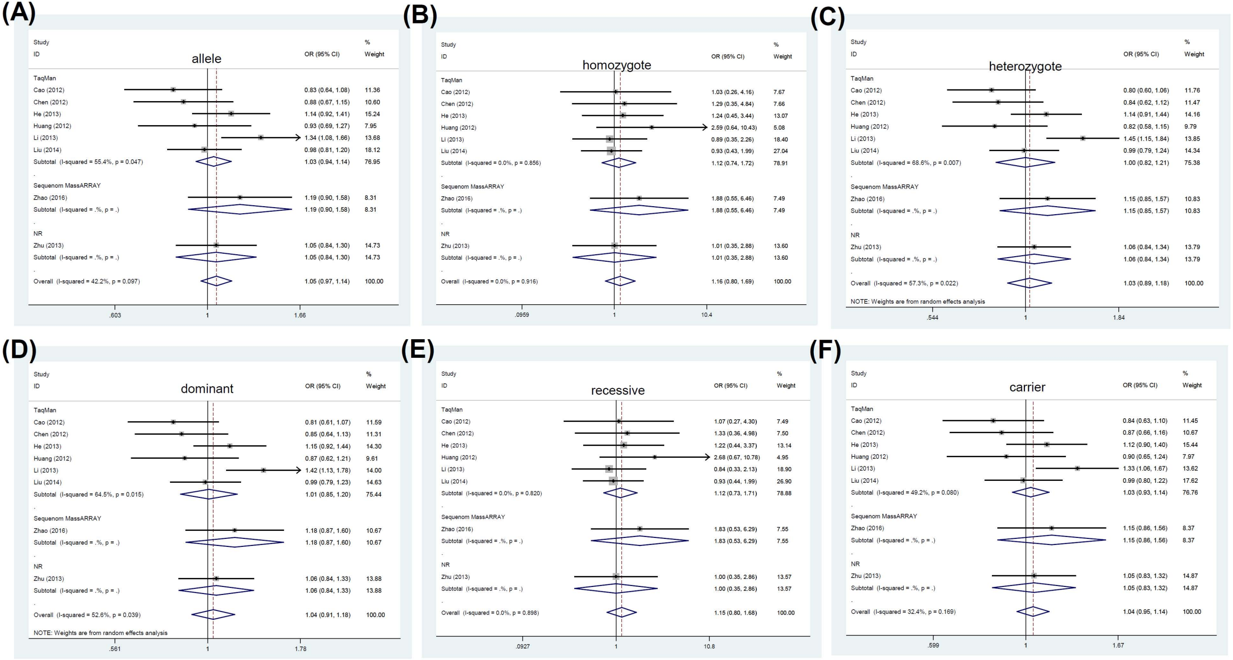

(A)
allele
Study ID
OR (95% CI)
% Weight
TaqMan
Cao (2012) 0.83 (0.64, 1.08) 11.36
Chen (2012) 0.88 (0.67, 1.15) 10.60
He (2013) 1.14 (0.92, 1.41) 15.24
Huang (2012) 0.93 (0.69, 1.27) 7.95
Li (2013) 1.34 (1.08, 1.66) 13.68
Liu (2014) 0.98 (0.81, 1.20) 18.12
Subtotal (I-squared = 55.4%, p = 0.047) 1.03 (0.94, 1.14) 76.95
Sequenom MassARRAY
Zhao (2016) 1.19 (0.90, 1.58) 8.31
Subtotal (I-squared = .%, p = .) 1.19 (0.90, 1.58) 8.31
NR
Zhu (2013) 1.05 (0.84, 1.30) 14.73
Subtotal (I-squared = .%, p = .) 1.05 (0.84, 1.30) 14.73
Overall (I-squared = 42.2%, p = 0.097) 1.05 (0.97, 1.14) 100.00
.603
1
1.66
(B)
homozygote
Study ID
OR (95% CI)
% Weight
TaqMan
Cao (2012) 1.03 (0.26, 4.16) 7.67
Chen (2012) 1.29 (0.35, 4.84) 7.66
He (2013) 1.24 (0.45, 3.44) 13.07
Huang (2012) 2.59 (0.64, 10.43) 5.08
Li (2013) 0.89 (0.35, 2.26) 18.40
Liu (2014) 0.93 (0.43, 1.99) 27.04
Subtotal (I-squared = 0.0%, p = 0.856) 1.12 (0.74, 1.72) 78.91
Sequenom MassARRAY
Zhao (2016) 1.88 (0.55, 6.46) 7.49
Subtotal (I-squared = .%, p = .) 1.88 (0.55, 6.46) 7.49
NR
Zhu (2013) 1.01 (0.35, 2.88) 13.60
Subtotal (I-squared = .%, p = .) 1.01 (0.35, 2.88) 13.60
Overall (I-squared = 0.0%, p = 0.916) 1.16 (0.80, 1.69) 100.00
.0959
1
10.4
(C)
heterozygote
Study ID
OR (95% CI)
% Weight
TaqMan
Cao (2012) 0.80 (0.60, 1.06) 11.76
Chen (2012) 0.84 (0.62, 1.12) 11.47
He (2013) 1.14 (0.91, 1.44) 14.16
Huang (2012) 0.82 (0.58, 1.15) 9.79
Li (2013) 1.45 (1.15, 1.84) 13.85
Liu (2014) 0.99 (0.79, 1.24) 14.34
Subtotal (I-squared = 68.6%, p = 0.007) 1.00 (0.82, 1.21) 75.38
Sequenom MassARRAY
Zhao (2016) 1.15 (0.85, 1.57) 10.83
Subtotal (I-squared = .%, p = .) 1.15 (0.85, 1.57) 10.83
NR
Zhu (2013) 1.06 (0.84, 1.34) 13.79
Subtotal (I-squared = .%, p = .) 1.06 (0.84, 1.34) 13.79
Overall (I-squared = 57.3%, p = 0.022) 1.03 (0.89, 1.18) 100.00
NOTE: Weights are from random effects analysis
.544
1
1.84
(D)
dominant
Study ID
OR (95% CI)
% Weight
TaqMan
Cao (2012) 0.81 (0.61, 1.07) 11.59
Chen (2012) 0.85 (0.64, 1.13) 11.31
He (2013) 1.15 (0.92, 1.44) 14.30
Huang (2012) 0.87 (0.62, 1.21) 9.61
Li (2013) 1.42 (1.13, 1.78) 14.00
Liu (2014) 0.99 (0.79, 1.23) 14.63
Subtotal (I-squared = 64.5%, p = 0.015) 1.01 (0.85, 1.20) 75.44
Sequenom MassARRAY
Zhao (2016) 1.18 (0.87, 1.60) 10.67
Subtotal (I-squared = .%, p = .) 1.18 (0.87, 1.60) 10.67
NR
Zhu (2013) 1.06 (0.84, 1.33) 13.88
Subtotal (I-squared = .%, p = .) 1.06 (0.84, 1.33) 13.88
Overall (I-squared = 52.6%, p = 0.039) 1.04 (0.91, 1.18) 100.00
NOTE: Weights are from random effects analysis
.561
1
1.78
(E)
recessive
Study ID
OR (95% CI)
% Weight
TaqMan
Cao (2012) 1.07 (0.27, 4.30) 7.49
Chen (2012) 1.33 (0.36, 4.98) 7.50
He (2013) 1.22 (0.44, 3.37) 13.14
Huang (2012) 2.68 (0.67, 10.78) 4.95
Li (2013) 0.84 (0.33, 2.13) 18.90
Liu (2014) 0.93 (0.44, 1.99) 26.90
Subtotal (I-squared = 0.0%, p = 0.820) 1.12 (0.73, 1.71) 78.88
Sequenom MassARRAY
Zhao (2016) 1.83 (0.53, 6.29) 7.55
Subtotal (I-squared = .%, p = .) 1.83 (0.53, 6.29) 7.55
NR
Zhu (2013) 1.00 (0.35, 2.86) 13.57
Subtotal (I-squared = .%, p = .) 1.00 (0.35, 2.86) 13.57
Overall (I-squared = 0.0%, p = 0.898) 1.15 (0.80, 1.68) 100.00
.0927
1
10.8
(F)
carrier
Study ID
OR (95% CI)
% Weight
TaqMan
Cao (2012) 0.84 (0.63, 1.10) 11.45
Chen (2012) 0.87 (0.66, 1.16) 10.67
He (2013) 1.12 (0.90, 1.40) 15.44
Huang (2012) 0.90 (0.65, 1.24) 7.97
Li (2013) 1.33 (1.06, 1.67) 13.62
Liu (2014) 0.99 (0.80, 1.22) 17.62
Subtotal (I-squared = 49.2%, p = 0.080) 1.03 (0.93, 1.14) 76.76
Sequenom MassARRAY
Zhao (2016) 1.15 (0.86, 1.56) 8.37
Subtotal (I-squared = .%, p = .) 1.15 (0.86, 1.56) 8.37
NR
Zhu (2013) 1.05 (0.83, 1.32) 14.87
Subtotal (I-squared = .%, p = .) 1.05 (0.83, 1.32) 14.87
Overall (I-squared = 32.4%, p = 0.169) 1.04 (0.95, 1.14) 100.00
.599
1
1.67

Figure S12

Figure S12. **Forest plot of subgroup analyses of *mTOR* rs2536 by genotyping method. (**A**) Allele; (**B**) homozygote; (**C**) heterozygote; (**D**) dominant; (**E**) recessive; (**F**) carrier.**

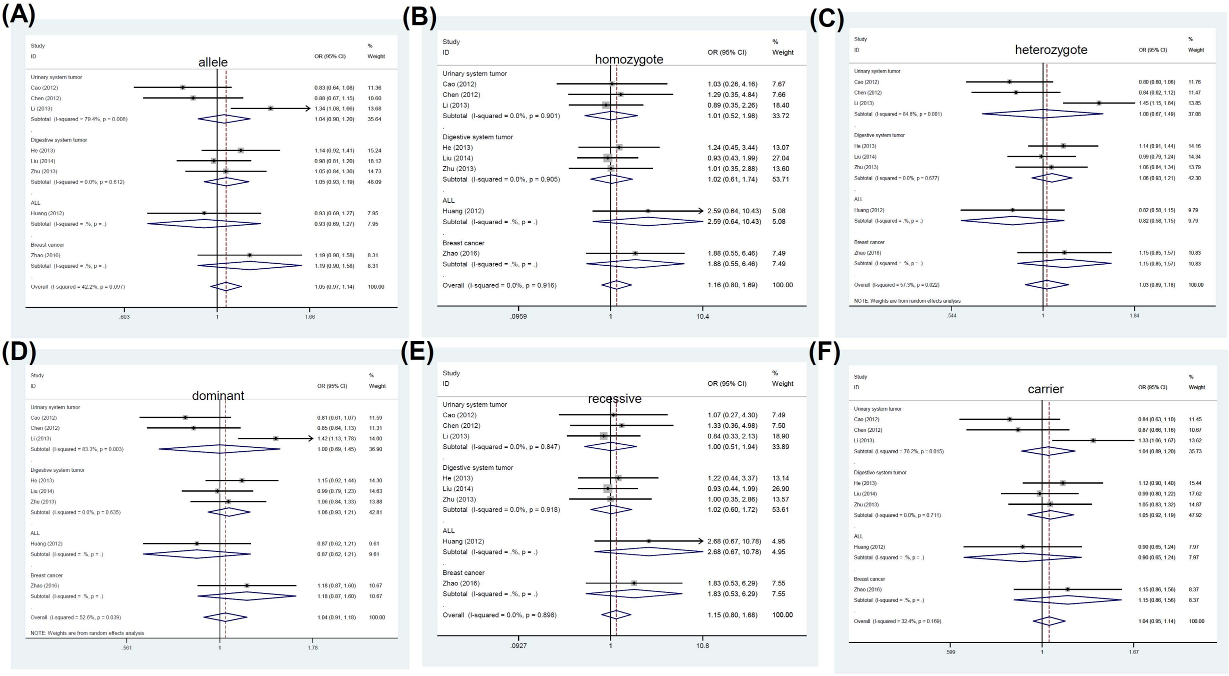
(A)
allele
Study ID | OR (95% CI) | % Weight
Urinary system tumor
Cao (2012) 0.83 (0.64, 1.08) 11.36
Chen (2012) 0.88 (0.67, 1.15) 10.60
Li (2013) 1.34 (1.08, 1.66) 13.68
Subtotal (I-squared = 79.4%, p = 0.008) 1.04 (0.90, 1.20) 35.64
Digestive system tumor
He (2013) 1.14 (0.92, 1.41) 15.24
Liu (2014) 0.98 (0.81, 1.20) 18.12
Zhu (2013) 1.05 (0.84, 1.30) 14.73
Subtotal (I-squared = 0.0%, p = 0.612) 1.05 (0.93, 1.19) 48.09
ALL
Huang (2012) 0.93 (0.69, 1.27) 7.95
Subtotal (I-squared = .%, p = .) 0.93 (0.69, 1.27) 7.95
Breast cancer
Zhao (2016) 1.19 (0.90, 1.58) 8.31
Subtotal (I-squared = .%, p = .) 1.19 (0.90, 1.58) 8.31
Overall (I-squared = 42.2%, p = 0.097) 1.05 (0.97, 1.14) 100.00
.603 1 1.66
(B)
homozygote
Study ID | OR (95% CI) | % Weight
Urinary system tumor
Cao (2012) 1.03 (0.26, 4.16) 7.67
Chen (2012) 1.29 (0.35, 4.84) 7.66
Li (2013) 0.89 (0.35, 2.26) 18.40
Subtotal (I-squared = 0.0%, p = 0.901) 1.01 (0.52, 1.98) 33.72
Digestive system tumor
He (2013) 1.24 (0.45, 3.44) 13.07
Liu (2014) 0.93 (0.43, 1.99) 27.04
Zhu (2013) 1.01 (0.35, 2.88) 13.60
Subtotal (I-squared = 0.0%, p = 0.905) 1.02 (0.61, 1.74) 53.71
ALL
Huang (2012) 2.59 (0.64, 10.43) 5.08
Subtotal (I-squared = .%, p = .) 2.59 (0.64, 10.43) 5.08
Breast cancer
Zhao (2016) 1.88 (0.55, 6.46) 7.49
Subtotal (I-squared = .%, p = .) 1.88 (0.55, 6.46) 7.49
Overall (I-squared = 0.0%, p = 0.916) 1.16 (0.80, 1.69) 100.00
.0959 1 10.4
(C)
heterozygote
Study ID | OR (95% CI) | % Weight
Urinary system tumor
Cao (2012) 0.80 (0.60, 1.06) 11.76
Chen (2012) 0.84 (0.62, 1.12) 11.47
Li (2013) 1.45 (1.15, 1.84) 13.85
Subtotal (I-squared = 84.8%, p = 0.001) 1.00 (0.67, 1.49) 37.08
Digestive system tumor
He (2013) 1.14 (0.91, 1.44) 14.16
Liu (2014) 0.99 (0.79, 1.24) 14.34
Zhu (2013) 1.06 (0.84, 1.34) 13.79
Subtotal (I-squared = 0.0%, p = 0.677) 1.06 (0.93, 1.21) 42.30
ALL
Huang (2012) 0.82 (0.58, 1.15) 9.79
Subtotal (I-squared = .%, p = .) 0.82 (0.58, 1.15) 9.79
Breast cancer
Zhao (2016) 1.15 (0.85, 1.57) 10.83
Subtotal (I-squared = .%, p = .) 1.15 (0.85, 1.57) 10.83
Overall (I-squared = 57.3%, p = 0.022) 1.03 (0.89, 1.18) 100.00
NOTE: Weights are from random effects analysis
.544 1 1.84
(D)
dominant
Study ID | OR (95% CI) | % Weight
Urinary system tumor
Cao (2012) 0.81 (0.61, 1.07) 11.59
Chen (2012) 0.85 (0.64, 1.13) 11.31
Li (2013) 1.42 (1.13, 1.78) 14.00
Subtotal (I-squared = 83.3%, p = 0.003) 1.00 (0.69, 1.45) 36.90
Digestive system tumor
He (2013) 1.15 (0.92, 1.44) 14.30
Liu (2014) 0.99 (0.79, 1.23) 14.63
Zhu (2013) 1.06 (0.84, 1.33) 13.88
Subtotal (I-squared = 0.0%, p = 0.635) 1.06 (0.93, 1.21) 42.81
ALL
Huang (2012) 0.87 (0.62, 1.21) 9.61
Subtotal (I-squared = .%, p = .) 0.87 (0.62, 1.21) 9.61
Breast cancer
Zhao (2016) 1.18 (0.87, 1.60) 10.67
Subtotal (I-squared = .%, p = .) 1.18 (0.87, 1.60) 10.67
Overall (I-squared = 52.6%, p = 0.039) 1.04 (0.91, 1.18) 100.00
NOTE: Weights are from random effects analysis
.561 1 1.78
(E)
recessive
Study ID | OR (95% CI) | % Weight
Urinary system tumor
Cao (2012) 1.07 (0.27, 4.30) 7.49
Chen (2012) 1.33 (0.36, 4.98) 7.50
Li (2013) 0.84 (0.33, 2.13) 18.90
Subtotal (I-squared = 0.0%, p = 0.847) 1.00 (0.51, 1.94) 33.89
Digestive system tumor
He (2013) 1.22 (0.44, 3.37) 13.14
Liu (2014) 0.93 (0.44, 1.99) 26.90
Zhu (2013) 1.00 (0.35, 2.86) 13.57
Subtotal (I-squared = 0.0%, p = 0.918) 1.02 (0.60, 1.72) 53.61
ALL
Huang (2012) 2.68 (0.67, 10.78) 4.95
Subtotal (I-squared = .%, p = .) 2.68 (0.67, 10.78) 4.95
Breast cancer
Zhao (2016) 1.83 (0.53, 6.29) 7.55
Subtotal (I-squared = .%, p = .) 1.83 (0.53, 6.29) 7.55
Overall (I-squared = 0.0%, p = 0.898) 1.15 (0.80, 1.68) 100.00
.0927 1 10.8
(F)
carrier
Study ID | OR (95% CI) | % Weight
Urinary system tumor
Cao (2012) 0.84 (0.63, 1.10) 11.45
Chen (2012) 0.87 (0.66, 1.16) 10.67
Li (2013) 1.33 (1.06, 1.67) 13.62
Subtotal (I-squared = 76.2%, p = 0.015) 1.04 (0.89, 1.20) 35.73
Digestive system tumor
He (2013) 1.12 (0.90, 1.40) 15.44
Liu (2014) 0.99 (0.80, 1.22) 17.62
Zhu (2013) 1.05 (0.83, 1.32) 14.87
Subtotal (I-squared = 0.0%, p = 0.711) 1.05 (0.92, 1.19) 47.92
ALL
Huang (2012) 0.90 (0.65, 1.24) 7.97
Subtotal (I-squared = .%, p = .) 0.90 (0.65, 1.24) 7.97
Breast cancer
Zhao (2016) 1.15 (0.86, 1.56) 8.37
Subtotal (I-squared = .%, p = .) 1.15 (0.86, 1.56) 8.37
Overall (I-squared = 32.4%, p = 0.169) 1.04 (0.95, 1.14) 100.00
.599 1 1.67

Figure S13

Figure S13. **Forest plot of subgroup analyses of *mTOR* rs2536 by cancer type. (**A**) Allele; (**B**) homozygote; (**C**) heterozygote; (**D**) dominant; (**E**) recessive; (**F**) carrier.**

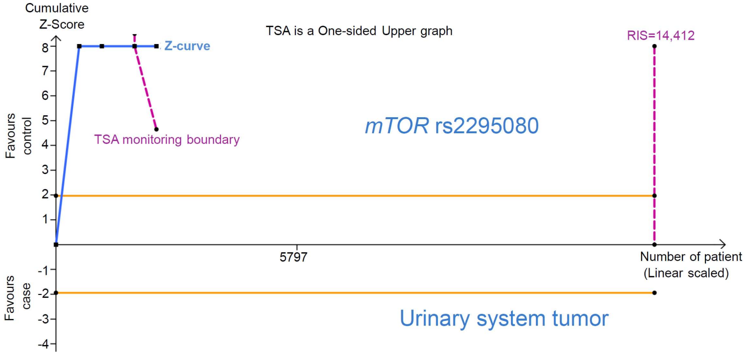
Cumulative
Z-Score
TSA is a One-sided Upper graph
RIS=14,412
Z-curve
Favours
control
TSA monitoring boundary
mTOR rs2295080
8
7
6
5
4
3
2
1
-1
-2
-3
-4
5797
Number of patient
(Linear scaled)
Favours
case
Urinary system tumor

Figure S14

Figure S14. **Trial sequential analysis of the association between *mTOR* rs2295080 and urinary system tumor risk via the dominant comparison.**

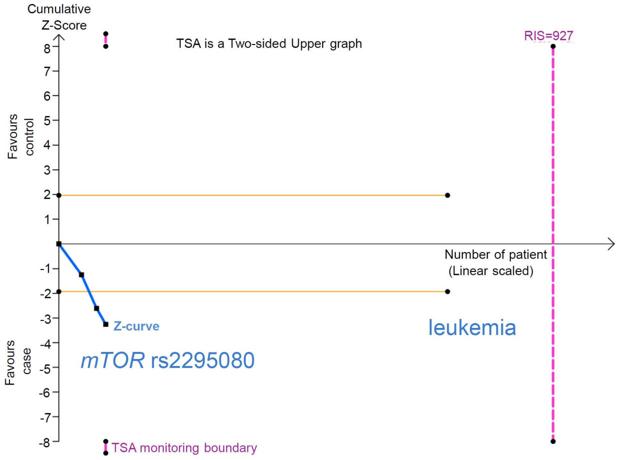
Cumulative
Z-Score
TSA is a Two-sided Upper graph
RIS=927
8
7
6
5
4
3
2
1
-1
-2
-3
-4
-5
-6
-7
-8
Favours control
Favours case
Number of patient
(Linear scaled)
Z-curve
leukemia
mTOR rs2295080
TSA monitoring boundary

Figure S15

Figure S15. **Trial sequential analysis of the association between *mTOR* rs2295080 and leukemia risk via the recessive comparison.**

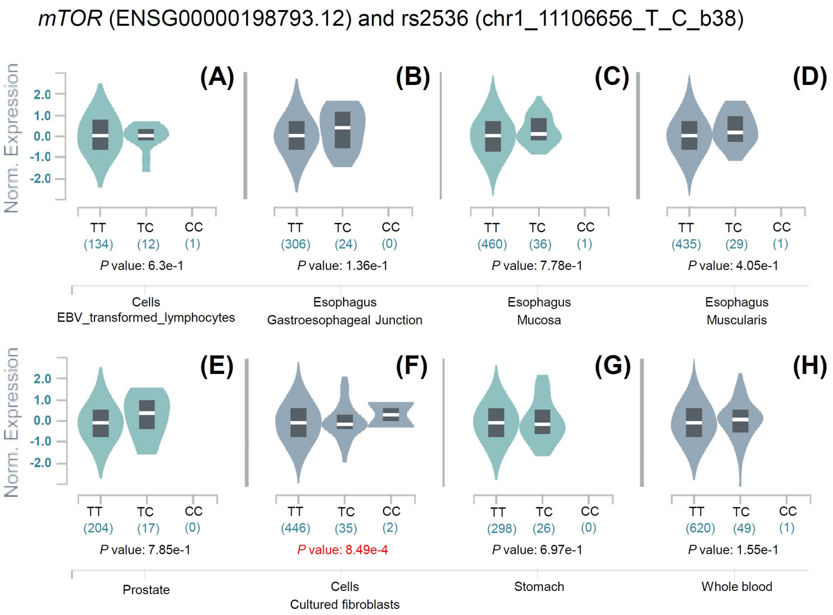

mTOR (ENSG00000198793.12) and rs2536 (chr1_11106656_T_C_b38)
Norm. Expression
2.0
1.0
0.0
-1.0
-2.0
(A)
(B)
(C)
(D)
TT (134) TC (12) CC (1)
P value: 6.3e-1
TT (306) TC (24) CC (0)
P value: 1.36e-1
TT (460) TC (36) CC (1)
P value: 7.78e-1
TT (435) TC (29) CC (1)
P value: 4.05e-1
Cells EBV_transformed_lymphocytes
Esophagus Gastroesophageal Junction
Esophagus Mucosa
Esophagus Muscularis
(E)
(F)
(G)
(H)
TT (204) TC (17) CC (0)
P value: 7.85e-1
TT (446) TC (35) CC (2)
P value: 8.49e-4
TT (298) TC (26) CC (0)
P value: 6.97e-1
TT (620) TC (49) CC (1)
P value: 1.55e-1
Prostate
Cells Cultured fibroblasts
Stomach
Whole blood

Figure S16

Figure S16. **eQTL analysis of *mTOR* rs2536 in selected cells or tissues within the GTEx database.** (**A**) **EBV_transformed_lymphocytes;** (**B**) **Esophagus_Gastroesophageal_Junction;** (**C**) **Esophagus_Mucosa;** (**D**) **Esophagus_Muscularis;** (**E**) **Prostate;** (**F**) **cultured_fibroblasts;** (**F**) **Stomach;** (**H**) **whole blood.**

Table S1. Database search terms.

**(1) PubMed database**			
**#1**	**#2**	**#3**	**#4**
((((((Neoplasms) OR Neoplasia) OR Neoplasias) OR Neoplasm) OR Tumors) OR Tumor) OR Cancer) OR Cancers	(((mTOR) OR Mammalian target of rapamycin) OR FRAP) OR FKBP12-rapamycin complex-associated protein	((((((((Polymorphism) OR Polymorphism, Genetic) OR Polymorphisms, Genetic) OR Genetic Polymorphisms) OR Genetic Polymorphism) OR Polymorphism (Genetics)) OR Polymorphisms (Genetics)) OR Polymorphisms) OR rs2536) OR rs2295080	(((((mTOR) OR Mammalian target of rapamycin) OR FRAP) OR FKBP12-rapamycin complex-associated protein)) AND (((((((Neoplasms) OR Neoplasia) OR Neoplasias) OR Neoplasm) OR Tumors) OR Tumor) OR Cancer) OR Cancers)) AND (((((((((Polymorphism) OR Polymorphism, Genetic) OR Polymorphisms, Genetic) OR Genetic Polymorphisms) OR Genetic Polymorphism) OR Polymorphism (Genetics)) OR Polymorphisms (Genetics)) OR Polymorphisms) OR rs2536) OR rs2295080)
**4,553,668**	**45,424**	**341,334**	**233**
**(2) Embase database**			
**#1**	**#2**	**#3**	**#4**
'neoplasms'/exp OR 'neoplasms' OR 'neoplasia'/exp OR 'neoplasia' OR 'neoplasias' OR 'neoplasm'/exp OR 'neoplasm' OR 'tumors'/exp OR 'tumors' OR 'tumor'/exp OR 'tumor' OR 'cancer'/exp OR 'cancer' OR 'cancers'/exp OR 'cancers'	'mtor'/exp OR 'mtor' OR 'mammalian target of rapamycin'/exp OR 'mammalian target of rapamycin' OR 'frap' OR 'fkbp12-rapamycin complex-associated protein'	'polymorphism'/exp OR 'polymorphism' OR 'polymorphisms' OR 'genetic polymorphisms' OR 'genetic polymorphism'/exp OR 'genetic polymorphism' OR 'rs2536' OR 'rs2295080'	#1 AND #2 AND #3

6,343,682	78,916	521,258	848
**(3) Cochrane**			
**#1**	**#2**	**#3**	**#4**
('mTOR') OR ('Mammalian target of rapamycin') OR ('FRAP') (Word variations have been searched)	('Polymorphism') OR ('Genetic Polymorphism') OR ('rs2536') OR ('rs2295080') (Word variations have been searched)	('Neoplasia') OR ('Neoplasm'') OR ('Tumor') OR ('Cancer') (Word variations have been searched)	#1 and #2 and #3
1,436	8,689	196,203	8
**(4) WANFANG**			
mTOR and Polymorphism and cancer			
25			

Table S2. Quality assessment score.

Study	Representativeness of case	Representativeness of control	Ascertainment of case	Control selection	Genotyping examination	HWE	Total sample size	Score
**Cao, 2012**	1	1	2	2	1	1	3	11
**Chen, 2012**	1	1	2	1	2	1	3	11
**Chen, 2019**	1	3	2	0	1	1	2	10
**He, 2013**	1	3	1	2	1	1	3	12
**Huang, 2012**	1	1	2	2	1	1	2	10
**Li, 2013**	1	3	2	1	1	1	3	12
**Liu, 2017**	1	1	2	1	1	1	3	10
**Liu, 2014**	1	1	0	2	2	1	3	10
**Qi, 2017**	1	1	2	2	0	1	3	10
**Wang, 2015**	1	1	2	2	1	1	3	11
**Wen, 2017**	1	3	2	2	2	1	3	14
**Xu, 2015**	1	1	2	2	1	1	3	11
**Xu, 2013**	1	1	2	2	2	1	3	12
**Zhao, 2017**	1	3	2	2	1	1	2	12
**Zhao, 2015**	1	1	2	2	1	1	2	10
**Zhao, 2016**	1	1	2	2	0	1	3	10
**Zhu, 2015**	1	3	2	2	1	1	3	13
**Zhu, 2013**	1	3	2	2	1	1	3	13
